# Oculomotor Function in Children and Adolescents with Autism, ADHD or Co-occurring Autism and ADHD

**DOI:** 10.1007/s10803-024-06718-3

**Published:** 2025-01-24

**Authors:** Elana J. Forbes, Jeggan Tiego, Joshua Langmead, Kathryn E. Unruh, Matthew W. Mosconi, Amy Finlay, Kathryn Kallady, Lydia Maclachlan, Mia Moses, Kai Cappel, Rachael Knott, Tracey Chau, Vishnu Priya Mohanakumar Sindhu, Alessio Bellato, Madeleine J. Groom, Rebecca Kerestes, Mark A. Bellgrove, Beth P. Johnson

**Affiliations:** 1 School of Psychological Sciences, Monash University, 18 Innovation Walk, Melbourne, VIC 3800 Australia; 2 Life Span Institute and Kansas Center for Autism Research and Training, The University of Kansas, 12610 Quivira Rd #270, Overland Park, KS 66213 USA; 3 School of Psychology, University of Southampton, Southampton, SO17 1PS UK; 4 Centre for Innovation in Mental Health, University of Southampton, Southampton, UK; 5 Institute for Life Sciences, University of Southampton, Southampton, UK; 6 School of Psychology, University of Nottingham, Semenyih, Malaysia; 7 Mind and Neurodevelopment Research Group, University of Nottingham, Semenyih, Malaysia; 8 School of Medicine, Academic Unit of Mental Health & Clinical Neurosciences, Institute of Mental Health, University of Nottingham, Triumph Road, Nottingham, NG7 2TU UK; 9 Department of Pediatrics, Monash University, Monash Children’s Hospital, Level 5, 246 Clayton Rd, Melbourne, VIC 3168 Australia

**Keywords:** Autism, ADHD, Neurodevelopment, Oculomotor Control, Endophenotype

## Abstract

Oculomotor characteristics, including accuracy, timing, and sensorimotor processing, are considered sensitive intermediate phenotypes for understanding the etiology of neurodevelopmental conditions, such as autism and ADHD. Oculomotor characteristics have predominantly been studied separately in autism and ADHD. Despite the high rates of co-occurrence between these conditions, only one study has investigated oculomotor processes among those with co-occurring autism + ADHD. Four hundred and five (*n* = 405; 226 males) Australian children and adolescents aged 4 to 18 years (M = 9.64 years; *SD* = 3.20 years) with ADHD (*n* = 64), autism (*n* = 66), autism + ADHD (*n* = 146), or neurotypical individuals (*n* = 129) were compared across four different oculomotor tasks: visually guided saccade, anti-saccade, sinusoidal pursuit and step-ramp pursuit. Confirmatory analyses were conducted using separate datasets acquired from the University of Nottingham UK (*n* = 17 autism, *n* = 22 ADHD, *n* = 32 autism + ADHD, *n* = 30 neurotypical) and University of Kansas USA (*n* = 29 autism, *n* = 41 neurotypical). Linear mixed effect models controlling for sex, age and family revealed that children and adolescents with autism + ADHD exhibited increased variability in the accuracy of the final saccadic eye position compared to neurotypical children and adolescents. Autistic children and adolescents demonstrated a greater number of catch-up saccades during step-ramp pursuit compared to neurotypical children and adolescents. These findings suggest that select differences in saccadic precision are unique to autistic individuals with co-occurring ADHD, indicating that measuring basic sensorimotor processes may be useful for parsing neurodevelopment and clinical heterogeneity in autism.

**Supplementary Information:**

The online version contains supplementary material available at 10.1007/s10803-024-06718-3.

Autism spectrum disorder (autism) and attention-deficit/hyperactivity disorder (ADHD) are highly prevalent, heterogeneous neurodevelopmental disorders, diagnosed in 2–4% and 5.9% of children, respectively (Faraone et al., 2021; Li et al., 2022). There is considerable clinical overlap between autism and ADHD, with 30–80% of autistic (Vivanti, 2020) individuals exhibiting ADHD symptomatology (Mayes et al., 2012; Reiersen & Todd, 2008), and 20–50% of individuals with ADHD having co-occurring autistic symptoms (Grzadzinski et al., 2016; Rommelse et al., 2010). Autism is characterized by difficulties with social communication, altered sensory processing, and restricted and repetitive patterns of behaviors and interests, whereas ADHD is defined by hyperactivity, impulsivity and inattention that may differ from societal expectations based on age (American Psychiatric Association, 2022). In the absence of validated biological markers to clinically identify autism or ADHD (Cortese et al., 2023), the diagnostic process and subsequent access to interventions are often protracted, relying heavily on multi-informant behavioral assessments and developmental histories. When children present with co-occurring autism and ADHD (autism + ADHD), diagnostic delays are particularly pronounced (Knott et al., 2024).

The high degree of clinical overlap between these conditions has also contributed to the considerable body of evidence for shared neurobiological and genetic underpinnings of autism and ADHD. Autism and ADHD are associated with various genetic and non-genetic etiologies (e.g., Modabbernia et al., 2017; Sciberras et al., 2017; Zhou et al., 2023). As such, research into endophenotypic traits—discrete, specific, quantifiable, biological markers that indicate the etiological likelihood of these conditions—have gained momentum (e.g., Balogh et al., 2022). Endophenotype research focuses on traits that act as intermediary processes between genetic causes and specific behavioral or clinical dimensions (Mosconi et al., 2010). While autism and ADHD are traditionally defined through categorical diagnoses in the Diagnostic and Statistical Manual of Mental Disorders (DSM-5) or International Classification of Diseases (ICD-11), research continues to explore potential objective markers that could aid in diagnosis, acknowledging the heterogeneous nature of these conditions (e.g., Waterhouse, 2022). Mapping endophenotypes across multiple levels (e.g., molecular, cellular, neural and neuropsychological) and the full range of neurodiversity can offer crucial insights into inherited traits contributing to clinical vulnerabilities and developmental differences that impact daily functioning. Findings of familial oculomotor differences in autism (Mosconi et al., 2010) supports the hypothesis that oculomotor control is a promising endophenotype for neurodevelopmental conditions (Mosconi et al., 2010).

Evidence from across three decades of research implicates oculomotor function as an important candidate endophenotype of autism and ADHD (Chamorro et al., 2022; Falck-Ytter et al., 2020; Mosconi et al., 2010; Pomè et al., 2023). The highly quantifiable, reproducible, heritable (Missitzi et al., 2013; Pinto et al., 2020) and observable nature of oculomotor metrics in autism (Bojanek et al., 2023; Mosconi & Sweeney, 2015) and ADHD (Chamorro et al., 2020) further promotes oculomotor function as a promising endophenotype across autism and ADHD. This approach has already been established in other areas of psychiatry including evidence that pursuit eye tracking abilities differentiate between bipolar disorder I and schizoaffective disorder (Lencer et al., 2015).

Although numerous studies have investigated oculomotor function in autism and ADHD (Chamorro et al., 2022; Johnson et al., 2016; Maron et al., 2021), very few have directly compared autistic individuals with and without co-occurring ADHD, limiting insights into whether oculomotor characteristics associated with autism may vary as a function of ADHD. To date, meta-analyses have demonstrated distinct oculomotor characteristics of autism, including (1) increased saccade amplitude variability (saccade dysmetria; i.e., overshooting or undershooting a saccadic target) when performing rapid eye movements to visual targets (i.e., visually guided saccades; VGS); (2) difficulty inhibiting reflexive saccades when performing inhibiting reflexive eye movements toward a target and instead looking in the opposite direction (i.e., anti-saccades; AS); and (3) impaired tracking of moving targets (i.e., smooth pursuit; Johnson et al., 2016). However, similar performances between autism and neurotypical groups have been observed across (1) saccade latency and basic saccade initiation during VGS; and (2) the ability to disengage and engage attention in gap-overlap tasks (Johnson et al., 2016). Limited research has investigated metrics of pursuit eye movement performance in autism, including pursuit latency and catch-up saccade frequency. Overall, these findings implicate altered functioning of bottom-up processing systems, particularly fundamental sensorimotor control in autism, including the ventral attentional network and cerebellar systems (McKinney et al., 2022). While autism is characterized by these bottom-up processing differences, meta-analyses of oculomotor performance in ADHD point to top-down processing difficulties, including: (1) higher intrusive saccades during fixation; (2) greater latency and more directional errors during AS; and (3) more anticipatory saccades during memory-guided saccades (Chamorro et al., 2022; Maron et al., 2021). However, similar performances between ADHD and neurotypical groups have been observed for: (1) latency and gain during VGS; (2) anticipatory saccades and latency during AS; and (3) latency, spatial accuracy, and memory recall errors during memory-guided saccades (Maron et al., 2021; Sherigar et al., 2023). Meanwhile, only a few studies have investigated smooth pursuit eye movements in ADHD (Maron et al., 2021; Sherigar et al., 2023), but the limited evidence to date suggests equivalent performance between ADHD and neurotypical groups in catch-up saccades and closed-loop gain. Overall, this points to altered functioning of top-down processing systems in ADHD, namely inhibitory, corrective and predictive control, as well as working memory (Amso & Scerif, 2015; Cortese et al., 2012; Hart et al., 2013). Significant knowledge gaps remain in understanding the oculomotor characteristics of individuals with co-occurring autism and ADHD. Recent work by Bellato et al. (2023) has begun to bridge these gaps by demonstrating slower saccadic reaction times during the gap-overlap task (including both baseline - which is equivalent to VGS - and overlap trials) in children with either or both conditions compared to those without autism or ADHD. However, no studies to date have systematically investigated shared and distinct oculomotor functions across the autism-ADHD spectra.

We evaluated the unique and shared oculomotor characteristics of autism and ADHD, and co-occurring autism + ADHD. We studied a range of oculomotor behaviors elicited using visually guided saccade (VGS), anti-saccade (AS), and smooth pursuit tasks. Based on previous findings (Johnson et al., 2016), we predicted the following: (1) autistic individuals would show reduced fundamental sensorimotor (i.e., bottom-up) control relative to individuals with ADHD and neurotypical controls, including increased latency to peak velocity of saccades, reduced gain (i.e., accuracy) of saccades, increased trial-to-trial variability of saccade gain, increased error and variability of final eye position during visually guided saccades, and reduced gain during smooth pursuit eye movements, and; (2) autistic individuals and individuals with ADHD each would show reduced inhibitory (i.e., top-down) control relative to neurotypical controls as evidenced by increased rates of anti-saccade errors during the AS task to be predominately implicated in ADHD as evidenced by anticipatory saccades on the AS task (Chamorro et al., 2022; Maron et al., 2021). The number of catch-up saccades and closed-loop gain during sinusoidal pursuit eye movements represent an intersection of both sensorimotor (i.e., bottom-up) processes with top-down processes including corrective and predictive control, respectively. Therefore, we expected both autistic and ADHD-only groups to demonstrate increased catch-up saccades and closed-loop gain relative to the neurotypical group. Finally, we hypothesized individuals with co-occurring ADHD (autism + ADHD) would demonstrate difficulties with oculomotor measures with underlying fundamental sensorimotor, inhibitory, corrective and predictive control processes (i.e., implicating *both* bottom-up and top-down networks), suggesting an additive model relative to autism-only and ADHD-only groups. Further, we anticipated that the autism + ADHD group would demonstrate greater reductions in sensorimotor control (e.g., increased latency to peak velocity), inhibitory control (e.g., increased anti-saccadic errors), corrective (i.e., more catch-up saccades during pursuit) and predictive control (i.e., increased closed-loop gain) compared to autism-only and ADHD-only groups. To examine the generalizability of our findings and their potential for clinical scaling, we also attempted to replicate our findings in multiple independent datasets acquired at separate institutions using similar oculomotor recording systems and stimulus paradigms.

## Methods

### Participants

Participants were 405 Australian children and adolescents aged 4–18 years old (226 Male; Mean Age = 9.64 years; *SD* = 3.20 years) who were enrolled in the Monash Autism-ADHD Genetics and Neurodevelopment (MAGNET) Project (henceforth, “MAGNET Cohort”; Table 1). Children and adolescents had a diagnosis of ADHD (*n* = 64), autism (*n* = 66) or co-occurring autism + ADHD (*n* = 146), or were neurotypical (*n* = 129) as confirmed by a best clinical estimate (BCE) approach by a registered psychologist. In short, the BCE protocol involved review of a child’s performance across a range of tasks, including the Autism Diagnostic Observation Schedule—2 (ADOS-2; Lord et al., 2012), Development and Wellbeing Assessment (DAWBA; Goodman et al., 2000), age-appropriate Wechsler intelligence scales, ADHD and autism symptom measures, and adaptive function measures. The BCE approach is recognized as the gold-standard in psychological diagnoses in research (Eijsbroek et al., 2024). Children and adolescents were excluded if they did not have enough data for a BCE (*n* = 47) or were deemed ineligible on review due to factors such as trauma history (*n* = 3). In some instances, multiple children and adolescents from the same family participated. Refer to Knott et al. (2021) for a detailed description of recruitment procedures, inclusion criteria and the BCE protocols. Ethics approval for the study was granted by Monash University Human Research Ethics Committee (HREC; CF16/1537–2016000806), Department of Education and Training Victoria HREC (2017_003570), and Monash Health HREC (RES-19-0000-372 A).

**Table 1:** Sample characteristics for MAGNET cohort ADHD, autism, autism + ADHD and neurotypical groups

	ADHD	Autism	ADHD + autism	NT	Whole sample
	*n =* 64	*n =* 66	*n =* 146	*n =* 129	*N =* 405
Child age (years)	10.10 (3.37)	9.51 (3.78)	9.51 (2.78)	9.62 (3.24)	9.64 (3.20)
Child sex					
Male	34	43	98	51	226
Female	30	23	48	78	179
FSIQ	100.48 (12.65)	98.29 (14.78)	98.36 (16.53)	107.05 (13.10)	101.44 (15.10)
ADOS-2					
Total	5.16 (4.42)	14.28 (5.19)	13.45 (4.61)	4.24 (3.97)	9.71 (6.40)
Comp	3.03 (2.39)	7.71 (2.18)	7.48 (2.00)	2.69 (2.24)	5.47 (3.19)
CPRS					
Hyp/Imp	72.23 (13.72)	59.66 (12.60)	75.15 (12.61)	52.51 (11.01)	64.95 (15.84)
Inatt	73.82 (11.74)	60.68 (11.08)	73.93 (11.15)	52.09 (10.30)	64.78 (14.75)
Total	75.63 (10.93)	61.56 (11.32)	76.42 (11.11)	52.69 (10.26)	66.30 (15.21)
SRS					
SCI	62.95 (11.83)	70.71 (11.87)	74.2 (12.11)	51.10 (9.63)	64.64 (14.99)
RRB	63.55 (12.24)	69.44 (14.07)	74.71 (12.41)	50.64 (9.38)	64.58 (15.54)
Total	63.81 (11.98)	72.13 (12.62)	75.8 (11.93)	51.24 (10.07)	65.64 (15.56)
Vineland-3					
ABC	85.69 (13.92)	79.88 (13.47)	77.78 (9.99)	99.12 (13.71)	85.31 (15.23)
Comm	86.88 (13.07)	83.47 (15.56)	80.67 (11.91)	99.01 (13.54)	87.21 (15.20)
Daily	87.9 (13.54)	82.13 (15.04)	80.66 (13.29)	99.41 (13.89)	87.26 (15.86)
Social	87.31 (17.12)	78.98 (15.59)	76.24 (13.24)	100.01 (12.31)	85.06 (17.23)

*Values* untransformed mean (Standard deviation), *ADHD* attention deficit/hyperactivity disorder, *Autism* autism spectrum disorder, *Autism + ADHD* co-occurring attention deficit/hyperactivity disorder and autism spectrum disorder, *NT* Neurotypical, *Child sex* frequency (percentage), *FSIQ* full scale intelligence quotient, *ADOS-2* autism diagnostic observation schedule–second edition, *Comp* ADOS-2 comparison score, *CPRS* Conners’ parent rating scale, *Hyp/Imp* hyperactive/impulsive, *Inatt* inattentive, *SRS* social responsiveness scale, *SCI* social communication index, *RRB* restricted repetitive behaviors, *Vineland-3* Vineland Adaptive Behavior Scale–Third Edition (Sparrow et al., 2016), *ABC* Vineland-3 adaptive behavior composite, *Comm* Vineland-3 communication subscale, *Daily* Vineland-3 daily living subscale

CPRS and SRS scores are age and gender matched *T*-scores

*Not all participants completed all measures. See Supplementary Table B1 for sample sizes summaries for each measure by group

### Measures

All clinical measures, symptom scales, and neurocognitive tasks followed the MAGNET testing protocol; all assessments were administered to all children and adolescents taking part in the project, regardless of diagnosis (Knott et al., 2021; See Supplementary Material C for additional details). Parents and caregivers provided information on their child’s sex (i.e., male or female) and age. See Table 1 for the means and standard deviations for Full-Scale Intelligence (FSIQ), ADOS-2, Social Responsiveness Scale (SRS-2), Conners’ Parent Rating Scale—Revised Long From (CPRS-RL) and Vineland Adaptive Behaviour Scale-Third Edition (VABS-3). The oculomotor measures were chosen for their robust test-retest reliability (Ettinger et al., 2003) and consistent associations with ADHD and/or autism (Chamorro et al., 2022; Johnson et al., 2016; Maron et al., 2021). All oculomotor methods are reported consistent with best-practice guidelines (Dunn et al. 2023; see Supplementary Table D).

#### Medication

Children and adolescents taking stimulant (e.g., methylphenidate, lisdexamfetamine, or dexamfetamine) or non-stimulant medication (e.g., atomoxetine) for ADHD were required to withdraw from their medication 48–72 h before completion of the eye tracking tasks (see Supplementary Material E for rates of medication use), however not for the cognitive assessment (e.g., WISC-V) or ADOS-2.

#### Wechsler Intelligence Assessments

FSIQ was determined using age-appropriate Weschler intelligence assessments. These included the Wechsler Preschool & Primary Scale of Intelligence—Fourth Edition (WPPSI-IV; Wechsler, 2012), Weschler Intelligence Scale for Children—Fifth Edition (WISC-V; Wechsler, 2016), Wechsler Adult Intelligence Scale—Fourth Edition (WAIS-IV; Wechsler, 2008), or Weschler Abbreviated Scale of Intelligence—Second Edition (WASI-II; Wechsler, 2011).

#### Symptom Measures

##### Social Responsiveness Scale—Second Edition (SRS-2)

The SRS-2 was used to measure autistic traits (Constantino, 2011). Comprising 65 items, caregivers rated their child’s behavior using a 4-point Likert scale. Scores from these items were aggregated to form indices for social communication, restricted and repetitive behaviors, and an overall total score. Raw scores underwent conversion to *T*-scores matched for age and gender, where higher scores indicate greater social difficulties and restricted and repetitive behaviors. The SRS is one of the most common screening instruments for autism (Moody et al., 2017).

##### Conners’ Parent Rating Scale—Revised Long from (CPRS-RL)

The 80-item CPRS-RL measures inattentive, hyperactive, and impulsive behaviors typical of ADHD (Conners et al., 1998). Scores obtained from a 4-point Likert scale were summed and transformed into *T*-scores matched for age and gender. The DSM-IV hyperactive/impulsive and inattentive subscales were used. It is recommended the CPRS-RL is included in comprehensive assessments of ADHD (Chang et al., 2016).

##### Vineland Adaptive Behaviour Scale—Third Edition (VABS-3)

The VABS-3 parent-rated questionnaire assesses adaptive behaviour, defined as the performance of daily activities required for personal and social sufficiency. This includes the individual’s ability to cope with environmental changes and learn new skills required for everyday living, and their level of independence (Sparrow et al., 2016). The scales of the VABS-3 are organised into the three broad domains of adaptive functioning specified by the American Association on Intellectual and Developmental Disabilities and by DSM-5 (Communication, Daily Living Skills, and Socialisation). The VABS-3 has high internal consistency (*a* = 0.96–0.99) and moderate to high concurrent validity (Pepperdine et al., 2018; Sparrow et al., 2016). The VABS is regarded as the most widely used adaptative behaviour instrument (Matson & Neal, 2009).

#### Oculomotor Tasks

Oculomotor data were collected using the EyeLink 1000, a non-invasive, desktop-mounted video-oculography system (sampling rate: 500 Hz; accuracy: 0.25–0.5 degrees). The participant’s head was stabilized using a headrest, positioned 84 cm away from the screen. The display screen was an LED LCD monitor (51 × 28.6 cm; 1920 × 1080 pixels). Eye positions were recorded for both eyes.

**Fig. 1:**
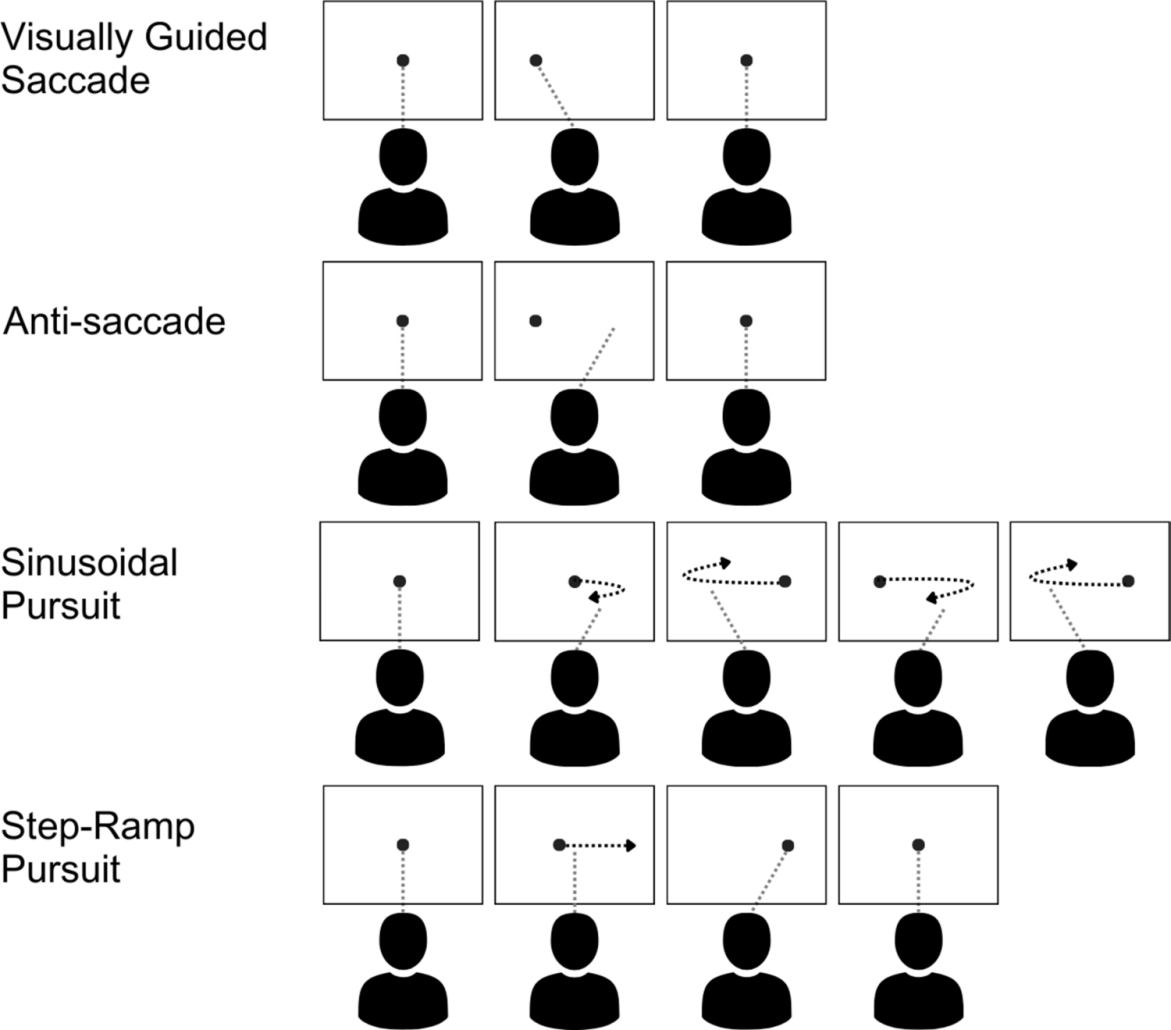
Schematic diagram of the four oculomotor tasks used. Full descriptions of these tasks are presented in Table 2 and derived outcome measures are presented in Table 3. Figure reproduced with permission (Maron et al., 2021)

Four oculomotor tasks were used in the MAGNET Cohort (see Fig. 1; Tables 2 and 3), including two saccade tasks: (1) visually guided saccades (VGS) and (2) anti-saccade (AS) tasks, and two smooth pursuit tasks: (3) sinusoidal pursuit and (4) step-ramp pursuit.

**Table 2:** Description of oculomotor tasks

Task	Description
Visually guided saccade task	Participants are instructed to fixate on a central target. A peripheral target then appears, prompting participants to make saccades (rapid eye movements) towards the new target. The central target then reappears, indicating the start of the next trial.
Anti-saccade task	Participants are instructed to fixate on a central target. A peripheral target then appears; participants are instructed to look in the *mirror opposite* location of the target. This requires participants to inhibit their reflexive eye movements towards a suddenly appearing target and instead make a voluntary eye movement in the opposite direction. The central target then reappears, indicating the start of the next trial.
Sinusoidal pursuit task	In this task, participants are required to track a target that moves in a horizontal sinusoidal or wave-like pattern across a screen. The goal is to maintain smooth and accurate eye movements to follow the target’s motion. Participants are instructed to follow a target with their eyes, whilst keeping their head still.
Step-ramp pursuit task	Participants are instructed to follow a target that moves smoothly across a screen, alternating between constant velocity segments (steps) and segments where the velocity increases or decreases gradually (ramps). Participants are instructed to follow a target with their eyes as accurately as possible, whilst keeping their head still.
Gap-overlap task*	We used the baseline condition from this task, which is equivalent to the visually guided saccade task for the purposes of confirmatory analyses. In the baseline condition, participants are instructed to fixate on a central fixation marker. This marker then turned off and a peripheral target appears.

Definitions according to Maron et al. (2021). Derived oculomotor measures from oculomotor tasks are defined in Table 3

*This task is relevant only to the Nottingham Cohort confirmatory analysis dataset

**Table 3:** Summary of oculomotor measures

Measure	Description
Visually guided saccade (VGS) task	
Relative time to peak velocity*	The time taken to reach peak velocity during a saccade, relative to the saccade amplitude (5^o^ or 10^o^).
Gain*	Gain quantifies the accuracy of eye movements, derived by dividing the eye movement position by the target position.
Gain variability*	The variability of gain.
Final eye position (FEP)*	The accuracy of the final eye position after a saccade, calculated as the amplitude of the initial saccade plus the amplitude of the corrected saccade.
FEP variability*	The variability in the final eye position.
Anti-saccade (AS) task	
Number of directional errors	Eye movements made to the wrong location, when the participant first looks at the target, rather than the mirror opposite location.
Number of anticipatory saccades	Anticipatory saccades are premature saccades made before target onset. Defined as eye movements that occur before the target appears, prior to the oculomotor system having time to initiate an exogenously driven saccade; default 100ms.
Step-ramp pursuit task	
Open-loop gain	The accuracy of the initial phase (first 100ms) of pursuit, calculated as the difference between eye position and target position. *Open-loop* refers to the movement being driven by sensory input relating to target motion (velocity and direction), without real-time feedback being yet available.
Sinusoidal pursuit task	
Closed-loop gain	Closed-loop pursuit is the period of pursuit after the initial open-loop period of pursuit. Closed-loop gain is defined as the difference between eye position and target position over the closed-loop period. *Closed-loop* refers to the fact that this type of movement relies primarily on current eye velocity and direction, target velocity and direction, feedback about performance (i.e. difference between eye and target motion), and predictions about future target position.
Both smooth pursuit tasks	
Number of catchup saccades	Saccades made in conjunction with pursuit eye movements, to correct for errors in eye position relative to target position

Definitions according to Maron et al. (2021)

*These measures were also derived from the baseline of the gap-overlap task administered in the Nottingham Cohort

##### Visually Guided Saccade Task

To measure saccade dynamics, a VGS task was administered. Prior to VGS testing, eye movements were calibrated using a three-point calibration (center, left and right). During testing, participants were instructed to fixate on a green cross (30 mm × 30 mm) in the center of the screen for a variable period of 1250ms or 1600ms. The central target then was extinguished and concomitantly a peripheral target (green cross) appeared for 1500ms at 5^o^ or 10^o^ to the right or left of center. The central fixation target then reappeared, indicating the beginning of the next trial. Participants completed 48 trials pseudorandomized across hemifields and target step amplitudes. The time to peak velocity, gain, and trial-wise gain variability of primary saccades were examined. We also measured the accuracy of final eye positions for each trial and their variability across trials for each individual (Table 3).

##### Anti-saccade Task

To measure inhibitory control of prepotent eye movements, an AS task was administered. Prior the AS task, eye movement was calibrated using a three-point calibration and participants were instructed to fixate on a green cross in the center of the screen. After a variable period of 1250ms or 1600ms, the central target extinguished, and a peripheral target (green cross) appeared 1500ms at 5^o^ or 10^o^ to the right or left of center. Stimulus characteristics were similar to the VGS task, but participants were instructed to look at the *mirror opposite* location of the peripheral target. That is, if the target was 5^o^ to the left of center, participants needed to look 5^o^ to the right of center. Participants were given up to three reminders to look in the mirror opposite location upon making any errors. The central fixation target then reappeared, indicating the beginning of the next trial. Six practice trials and 48 test trials followed calibration, with the target going left or right in an equal number of trials in a randomized order. The rate of anti-saccades and saccades made prior to the appearance of the peripheral target (anticipatory saccades) were examined (Table 3).

##### Sinusoidal Pursuit Eye Movement Task

To measure smooth pursuit eye movements, sinusoidal and step-ramp pursuit tests were administered. Prior to the sinusoidal pursuit task, eye movement was calibrated using a five-point calibration (right, left, center, top and bottom). During the sinusoidal pursuit test, a circular stimulus (10 mm in diameter) appeared on the screen for a 1000ms fixation period before moving left to right in a continuous, sinusoidal, motion. Peak-to-peak amplitude was 10° either side of center, slowing as it reached the turning point, with a 250 ms pause at each end. The stimulus moved at a velocity alternating between 6º/s and 18º/s to allow for a slow/fast condition. The stimulus varied in color to sustain the participant’s attention. Participants observed 15 oscillations across two blocks per fast/slow condition, with the target going left or right in an equal number of trials in a randomized order. See Table 3 for derived outcomes.

##### Step-Ramp Pursuit Eye Movement Task

During the step-ramp pursuit test, a circle appeared for a 1000ms fixation period the stimulus (circle) and then stepped 3° to the right or left of center (i.e., step phase), before moving continuously in the opposite direction in a trapezoidal waveform (i.e., ramp phase) for 555ms or 1670ms. The circle disappeared after reaching 10° for a 1500ms period of fixation, before reappearing in the center for the next trial. The circle was either inert or dynamic and target velocity alternated between 6°/s (slow condition) and 18°/s (fast condition) to create four alternating conditions: dynamic/slow, dynamic/fast, inert/slow and inert/fast. Two blocks of 16 trials each were presented, with the target going left or right in an equal number of trials in a randomized order. See Table 3 for derived outcomes.

### Procedure

#### Cohorts for Post-hoc Confirmatory Analyses

Confirmation of the study findings was attempted through analyses of datasets from The University of Nottingham (henceforth, “Nottingham Cohort”) and The University of Kansas (henceforth, “Kansas Cohort”), which included a partially overlapping testing protocol, comprising: (1) a gap-overlap task completed by the Nottingham Cohort (used as a VGS comparison) and (2) VGS and AS tasks completed by the Kansas Cohort.

#### Nottingham Cohort

##### Participants

The Nottingham Cohort comprised 101 children and adolescents aged 7–15 years from the UK (M = 10.78 years, *SD* = 2.04; *n* = 17 autism, *n* = 22 ADHD, *n* = 32 autism + ADHD, *n* = 30 neurotypical; Bellato et al., 2023; see Supplementary Table K1 for cohort characteristics). Participants who were diagnosed with or under clinical assessment for autism and/or ADHD were recruited from local support groups or were referred by health practitioners in local health or special educational services in Nottinghamshire (UK). Neurotypical participants were recruited from local schools and from a database of volunteers held by the School of Psychology, University of Nottingham UK. Participants were not excluded if they had a co-occurring diagnosis of mental health or behavioral conditions (including anxiety, depression, oppositional defiant or conduct disorder), or intellectual disability (i.e., IQ < 70). Children recruited as neurotypical controls were not included in the study if they were siblings of a child with a pre-existing diagnosis of autism, ADHD or any other ICD-10/DSM-5 psychiatric diagnoses. The Nottingham Cohort completed a gap-overlap task as part of a larger testing battery (including other eye-tracking and EEG tasks; Bellato et al., 2023) defined in Table 2. Parents and teachers completed the Conners’ Rating Scales (CRS-3) and Social Communication Questionnaire (SCQ). The ADOS-2 and WASI-II were administered to children and adolescents (see Supplementary Table K1). Children and adolescents were required to withdraw from stimulant medication for ADHD for at least 24 h before oculomotor testing (see Supplementary Tables K2 and K3), however not for the cognitive assessment (e.g., WISC-V) or ADOS-2.

##### Oculomotor Procedure

EyeLink 1000 was also used for the Nottingham Cohort (sampling rate: 500 Hz; accuracy: 0.25–0.5 degrees). Participants sat at an average distance of 60 cm from the display, which is the range recommended by EyeLink. Rather than using a chinrest, a sticker placed on the participant’s forehead was used to monitor their distance.

Participants in the Nottingham Cohort completed a gap-overlap task, of which the baseline condition of this task is comparable to the VGS test administered to the MAGNET Cohort (see Table 3). However, the stimuli and protocol used in the Nottingham Cohort were different from those used in the MAGNET Cohort. A nine-point calibration was used (center and eight peripheral points). During this task, the central stimulus was a color-filled circle, with a white cross in the middle, positioned in a dark grey background. The central stimulus expanded and contracted at regular intervals (500ms), until the EyeLink detected that the participant had fixated on the central marker for 1000ms. A peripheral stimulus then appeared for a variable duration of 500ms to 1500ms before a blank screen was presented and a new trial started. The task consisted of 12 blocks of 7 trials each, divided by video breaks of 6s duration, leading to a total of 84 task trials. The order of presentation of trials was randomized.

#### Kansas Cohort

##### Participants

The Kansas Cohort comprised 29 autistic and 41 neurotypical children and adolescents from the USA aged 5–18 years (M = 13.00 years, *SD* = 2.82). Autistic participants were recruited through patient registries including individuals seen for autism diagnostic testing at the University of Kansas Medical Center clinics. Diagnoses of autism were confirmed for the research study using the Autism Diagnostic Interview-Revised (ADI-R; Lord et al., 2012), the ADOS-2 (Lord et al., 2012), and expert clinical opinion based on DSM-5 criteria (American Psychiatric Association, 2022).

Neurotypical participants were recruited through a neurotypical research database at the University of Kansas and through fliers in the community. Neurotypical participants were excluded if they scored ≥ 8 on the Social Communication Questionnaire (SCQ; Lord et al., 2012), had a history of psychiatric illness, had a first-degree relative with a major psychiatric illness, or had a first- or second-degree relative with autism. Participants with a full-scale IQ < 70 based on the Wechsler Abbreviated Scale of Intelligence-II (WASI-II; Wechsler, 2011) were excluded from the study. All participants had normal or corrected visual acuity of 20/40 or better and were free of known genetic disorders associated with autism, history of head injury, or current use of psychotropic medications including antipsychotics, benzodiazepines, or anti-seizure medications.

See Supplementary Table L1 for full characteristics of the Kansas Cohort. The Kansas Cohort completed both VGS and AS tasks. Testing was completed across two to three visits. Autistic participants completed the ADOS-2, ADI-R and WASI-II during an initial visit. Oculomotor tasks were completed at a second visit. Visits were anywhere from 2 to 6 h, including breaks as needed. Participants were not asked to withdraw from any medications during their participation (Supplementary Table L3). Study procedures were approved by the University of Kansas Medical Center (KUMC) IRB.

##### Oculomotor Procedure

Again, EyeLink 1000 was used to record eye movements in the Kansas Cohort (sampling rate: 500 Hz; accuracy: 0.25–0.5 degrees). Participants completed VGS and AS tasks positioned 61 cm from a 27-inch LCD monitor (resolution: 2560 × 1440, refresh rate: 144 Hz). A chinrest was used to minimize head movement.

For both the VGS and AS tasks, participants fixated on a centrally located white crosshair for a variable amount of time (1.5–2 s) at the start of each trial. As the central fixation disappeared, a peripheral stimulus (i.e., white circle, 0.3° in diameter) appeared pseudorandomly at + 12° or 24° of visual angle for 1.5s. 15 trials for each of the four stimulus locations (60 total trials) were completed. In the VGS task, participants were instructed to look toward peripheral stimuli as quickly as possible. In the AS task, participants were instructed to inhibit saccades towards peripheral stimuli and instead make volitional saccades to the mirror location (i.e., anti-saccades). After 1.5s, the stimulus was extinguished. During the AS task, a green correction light then appeared in the mirror location. No age limitations were applied for participation in the AS task. In both tasks, the task was discontinued after the first block of (30) trials if it was apparent that the participant could not understand the instructions.

#### Oculomotor Data Processing

Oculomotor data from all cohorts underwent pre-processing using custom scripts developed by Langmead et al. (2024) in R and MATLAB, which were developed and validated using data from complex neurodevelopmental and paediatric populations, with reliability of the algorithm validated against manual cleaning processes from two independent sites. Langmead et al. (2024) reports inter-rater reliability between the automated pre-processing pipeline and manual cleaning with Cohen’s kappa values for inclusion/exclusion agreement of MAGNET data, showing κ = 0.70 (95% CI [0.69, 0.71], *p* < .001) for VGS trials and κ = 0.73 (95% CI [0.71, 0.75], *p* < .001) for AS trials. Inter-rater reliability was limited by errors in the manual processing of data, suggesting that automated processing is a more reliable and valid approach to pre-processing oculomotor data (Langmead et al., 2024). Summaries of outcomes derived from each oculomotor task are provided in Table 3. This automated pre-processing pipeline was applied to MAGNET, Nottingham and Kansas datasets, discussed further below.

### Statistical Analysis

#### MAGNET Cohort

Where the assumptions for classification as missing at random were met, multiple imputation was undertaken (Enders, 2017) using the fully conditional specification method (van Buuren, 2007). As only 116 participants had valid data for the AS task (see Supplementary Material F), multiple imputation was not undertaken for this outcome.

We imputed data using multiple imputation and results from analyses of imputed datasets were averaged to achieve a pooled result for each analysis. While as few as 5 multiple imputations are recommended (Enders, 2010), 50 imputations were run to ensure standard errors were small due to the smaller sample size and outliers were retained in the final dataset following outlier sensitivity analyses. Means and standard deviations for all transformed and imputed oculomotor task measures can be found in Supplementary Table A1.

A series of linear mixed effect (LME) models were used to evaluate differences in oculomotor function outcomes across ADHD, autism, autism + ADHD, and neurotypical groups. ANOVAs with target direction (saccade tasks) and amplitude (saccade tasks) or velocity (pursuit tasks) as the within-subjects variable and Diagnostic Group as the between-subjects variable revealed no significant interaction between group and these oculomotor dependent variables (DVs). Therefore, all oculomotor measures were collapsed across direction, amplitude and/or velocity to be entered into LME models as is standard for these measures in the developmental psychopathology literature (e.g., Johnson et al., 2012). Prior to conducting LME, linear regression models were constructed for each oculomotor variable to control for covariates: sex, age, age^2^, age^3^, age^4^, sex*age^2^, sex*age^3^, sex*age^4^. Unstandardized regression residuals were correlated with covariates at 0.00. The LME for each oculomotor variable then included the Diagnostic Group as a fixed effect, the unstandardized residual of the relevant linear regression model as a covariate, and Family as a random effect to control for the inclusion of siblings. The number of participants per level 2 group (i.e., Family) was deemed to be less important for level 1 (individual level) estimates (Garson, 2019). Fixed effects were estimated using the restricted maximum likelihood (REML) method, which is appropriate for estimating variance components in mixed-effect models and robust to unbalanced designs, small sample sizes and certain violations of normality, in order to compute the degrees of freedom and an *F*-statistic (Garson, 2019). Intraclass Correlation Coefficient (ICC), Log-likelihood, Akaike information criterion (AIC), and Bayesian information criterion (BIC) fit statistics for the Diagnostic Group LME models can be found in Supplementary Material G.

As multiple imputation yields biased estimates where data are missing not at random, auxiliary variables were incorporated to transform the missing data mechanism into a missing at random pattern (Enders, 2010; Hardt et al., 2012). Data were screened for invalid responses and transformed to approach a normal distribution prior to multiple imputation (MI) to uphold the assumptions of Markov Chain Monte Carlo procedures (Lee & Carlin, 2010; Supplementary Material H). As LME is regarded as robust against non-normality (Schielzeth et al., 2020), analyses proceeded without inspection of the normality of residuals. Additionally, non-linear (e.g., logarithmic) transformations change the correlations between variables, so a conservative approach is warranted. Outliers were removed using a conservative approach based on exceeding 2.2 interquartile range that is less sensitive to skewed distributions (Hoaglin et al., 1986) following multiple imputation and outlier sensitivity analyses conducted (Thabane et al., 2013). Results did not differ with outliers removed, and as such outliers were retained in the final model to uphold the most conservative approach. A Benjamini–Hochberg correction was employed to control the false discovery rate for all main effects at the DV level (Benjamini & Hochberg, 1995), resulting in adjusted alpha values of *p* < .030 for FEP variability and *p* < .031 for Step-Ramp Number of catch-up saccades. All other adjusted alpha values were *p* < .033 − .048. Significant main effects were then investigated, and relevant pairwise comparisons underwent Bonferroni correction. We report effect sizes for all group comparisons using Cohen’s *d*. We completed analyses in IBM SPSS Statistics (Version 29.0.1.0). Figures were generated in GraphPad Prism (Version 10.2.0).

#### Confirmatory Datasets

The same data cleaning methods as above were applied to the Nottingham and Kansas cohorts. No data were missing in the Nottingham Cohort nor Kansas Cohort AS data. As 16% of the Kansas VGS data were missing, multiple imputations were not applied to prevent biased imputations (Bennett, 2001). Data were screened for invalid responses, and outliers (Hoaglin et al., 1986) were winsorised as 3 SD following outlier sensitivity analyses. ANOVAs with target direction (both cohorts) and amplitude (Kansas Cohort) as the within-subjects variable and Diagnostic Group as the between-subjects variable were conducted. In the Kansas Cohort, significant interactions between Diagnostic Group and the direction and velocity conditions of the FEP variability measure were identified. No significant interaction between Diagnostic Group and oculomotor measures were otherwise identified, and all other variables were subsequently collapsed across conditions. Separate linear regressions were conducted for each oculomotor variable to control for covariates: sex, age, age^2^, age^3^, age^4^, sex*age^2^, sex*age^3^, sex*age^4^. The regression model for FEP variability in the Kansas Cohort additionally controlled for direction and velocity conditions. Unstandardized regression residuals were correlated with covariates at 0.00. As participants were not related in either cohort, the final LME model investigated diagnosis as a fixed effect, and regression residuals were entered as covariates. Outcome variables were oculomotor measures from the four oculomotor tasks: (1) VGS task: relative time to peak velocity, gain, gain variability, FEP, and FEP variability; (2) AS task: number of directional errors and number of anticipatory saccades; (3) Step-ramp task: open-loop gain and number of catch-up saccades; and (4) Sinusoidal pursuit task: closed-loop gain and number of catch-up saccades.

#### Post-hoc Comparisons of Cohort Characteristics

Chi-square tests and a one-way ANOVA were conducted to investigate group characteristics across the cohorts, being sex, age and Diagnostic Group.

## Results

### MAGNET Cohort

**Fig. 2:**
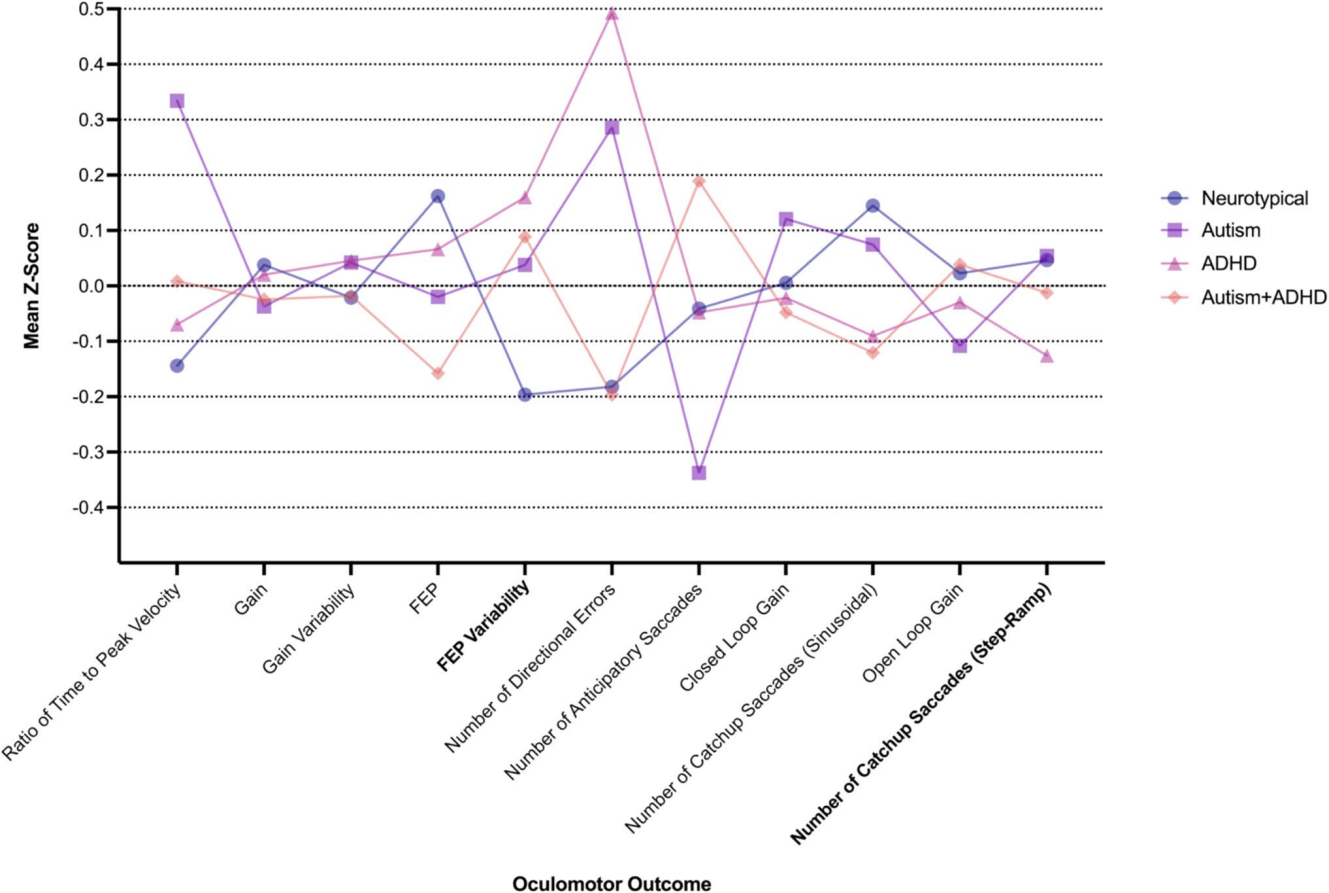
Oculomotor outcomes by diagnostic group. FEP Final Eye Position, ADHD Attention Deficit/Hyperactivity Disorder. Bold = significant main effect for diagnostic group following Benjamini Hochberg correction. Transformed and imputed data are presented. Here, lower scores are indicative of a more accurate performance

Mean *Z*-scores based on the current sample’s oculomotor performances are plotted in Fig. 2. The gain, closed loop gain, open loop gain, and the inverse-transformed relative time to peak velocity outcomes were inversely scored prior to *z*-score conversion so that lower scores on all measures were indicative of better performance. LME model statistics for the main effects of Diagnostic Group are presented in Supplementary Material I.

### Visually Guided Saccade task

All LME findings are reported in Supplementary Material I. The FEP variability outcome showed a main effect of Diagnostic Group (*F*(3,400) = 3.38, *p* = .025, *d =* 0.316). Pairwise comparisons (Supplementary Material J) revealed that children and adolescents with autism + ADHD had a significantly greater FEP variability than neurotypical children and adolescents (*p* = .002, *d =* 0.035). The autism + ADHD group showed no difference from the ADHD-only or autism-only groups. Main effects for Diagnostic Group were not observed for the relative time to peak velocity (*F*(3,400) = 1.74, *p* = .249, *d =* 0.227), gain (*F*(3,400) = 0.73, *p* = .576, *d =* 0.147), gain variability (*F*(3,400) = 2.50, *p* = .084, *d* = 0.272), nor FEP (*F*(3,400) = 0.92, *p* = ..457, *d =* 0.165).

### Anti-saccade Task

We did not observe statistically significant main effects for Diagnostic Group for the number of directional errors (*F*(3,111) = 0.36, *p* = .784, *d* = 0.194), or for the number of anticipatory saccades (*F*(3,111 = 0.33, *p* = .804, *d* = 0.186) outcomes.

### Sinusoidal Pursuit Task

We did not observe statistically significant main effects for Diagnostic Group for closed loop gain (*F*(3,400) = 2.94, *p* = .092, *d* = 0.295) or the number of catch-up saccades (*F*(3,400) = 3.55, *p* = .034, *d* = 0.324).

### Step-Ramp Pursuit Task

The number of catch-up saccades showed a statistically significant main effect of Diagnostic Group (*F*(3,400) = 3.09, *p* = .027, *d* = 0.302). Post-hoc pairwise comparisons (Supplementary Material J) revealed that autistic children and adolescents had a significantly greater number of catch-up saccades than neurotypical children and adolescents (*p* = .015, *d* = 0.035). The autistic group were not statistically different from the ADHD-only or autism + ADHD groups. There was no statistically significant main effect for Diagnostic Group for the open loop gain (*F*(3,400) = 2.94, *p* = .092, *d* = 0.295).

#### Post-hoc Confirmatory Analyses

##### Nottingham Cohort

We did not observe statistically significant main effects in the Nottingham Cohort for any of the variables (Supplementary Table K4): Relative time to peak velocity (*F*(3,96) = 2.34, *p* = .078, *d* = 0.334); Gain (*F*(3,96) = 0.64, *p* = .594, *d* = 0.174); Gain variability (*F*(3,96) = 0.83, *p* = .482, *d* = 0.199); FEP (*F*(3,96) = 0.99, *p* = .398, *d* = 0.217); FEP variability (*F*(3,96) = 0.94, *p* = .427, *d* = 0.211).

##### Kansas Cohorts

LME investigations in the Kansas Cohort are reported in Supplementary Table L4 and L5. We observed a statistically significant main effect of the number of directional errors (*F*(1,55) = 5.81, *p* = .019, *d* = 0.452), such that the autistic group demonstrated a greater number of directional errors on the AS task than neurotypical participants (*p* = .019, *d* = −0.624). We did not observe any other statistically significant main effects: relative time to peak velocity (*F*(1,55) = 4.51, *p* = .036, *d* = 0.398); gain (*F*(1,55) = 1.22, *p* = .273, *d* = 0.207); gain variability (*F*(1,55) = 2.69, *p* = .107, *d* = 0.307); FEP (*F*(1,55) = 0.19, *p* = .669, *d* = 0.082); FEP variability (*F*(1,55) = 5.25, *p* = .026, *d* = 0.429); number of anticipatory saccades (*F*(1,67) = 0.17, *p* = .680, *d* = 0.077).

#### Post-hoc Comparisons of Cohort Characteristics

Chi-square tests of independence showed a significant association between sex and cohort χ²(4, *N* = 575) = 356.54, *p* < .001 and between Diagnostic Group and cohort χ²(6, *N* = 575) = 70.62, *p* < .001. A one-way ANOVA was conducted to compare the effect of cohort on age. There was a significant effect of cohort on age, *F*(2,572) = 39.53, *p* < .001. Post-hoc Bonferroni-corrected comparisons revealed that the mean age of the MAGNET Cohort (*M* = 9.64 years) was significantly lower than both the Nottingham Cohort (*M* = 10.78 years), *p* = .002, with a mean difference of −1.14 years (95% CI [−1.94, −0.35]), and the Kansas Cohort (*M* = 13.00 years), *p* < .001, with a mean difference of −3.37 years (95% CI [−4.30, −2.43]). The Nottingham Cohort also had a significantly lower mean age than the Kansas Cohort, *p* < .001, with a mean difference of −2.22 years (95% CI [−3.34, −1.11]).

## Discussion

This study sought to characterize oculomotor function across autistic, ADHD, autism + ADHD and neurotypical groups of children and adolescents. Our results showed that children and adolescents in the MAGNET Cohort with autism + ADHD demonstrated the greatest variability in FEP during VGS compared to neurotypical children and adolescents. Autistic individuals demonstrated a greater number of catch-up saccades during the step-ramp pursuit task than neurotypical children and adolescents.

Children and adolescents with autism + ADHD exhibited greater variability in their FEP during the VGS task compared to neurotypical children and adolescents. Interestingly, a significant effect was not observed for the ADHD-only nor autism-only groups compared to neurotypical individuals, nor were any differences observed between autism + ADHD and autism-only or ADHD-only groups. This increased variability in FEP implicates key sensorimotor (i.e., bottom-up) circuits involved in saccade control and precision. The initial ballistic component of saccades relies on feedforward control through the superior colliculus (SC) and brainstem premotor circuits (Quaia et al., 1999). However, achieving a precise FEP requires intact feedback-dependent mechanisms, particularly involving the cerebellum. The cerebellar vermis lobules VI-VII and fastigial nucleus play a crucial role in monitoring and adjusting saccade amplitude through feedback from the brainstem, helping to minimize endpoint variability (Johnson et al., 2012; Mosconi et al., 2010). Greater FEP variability suggests disruption in this cerebellar-brainstem feedback loop that normally ensures saccade accuracy. Further, the anterior cingulate cortex (ACC) may also contribute to saccade error monitoring and correction (Thakkar et al., 2008). Neuroimaging evidence indicates atypical ACC activation during saccades in autism (Agam et al., 2010), which could impact online monitoring of saccade accuracy. Increased FEP variability has most consistently been identified as compromised in autism, however, there has been insufficient evidence to conclude whether these systems are affected in ADHD to date (Johnson et al., 2016; Maron et al., 2021; Sherigar et al., 2023). However, individuals with ADHD demonstrate a higher variability in their motor responses, in general, compared to neurotypical peers, which has been linked to atypical frontal-subcortical functioning (Castellanos et al., 2008; Castellanos et al., 2008; Suskauer et al., 2008). The lack of significant differences between the autism + ADHD group and autism-only or ADHD-only groups may be attributable to variability in these clinical groups masking possible differences. This effect was not replicated in the Nottingham Cohort, with no significant findings identified. This may be attributable to differences in testing stimuli, protocol (e.g., no use of a chinrest, which may have increased susceptibility to movement), statistically significant age and sex difference between cohorts, and/or the relatively smaller autism group in this cohort.

Our second finding revealed that autistic children and adolescents exhibited more catch-up saccades during the step-ramp pursuit task compared to neurotypical participants, with no significant differences observed in the ADHD-only or autism + ADHD groups compared to neurotypical or autistic individuals. The increased frequency of catch-up saccades in autistic participants suggests reduced saccadic accuracy and/or difficulty maintaining target tracking, specifically in matching eye velocity to target velocity. These findings implicate both sensorimotor (bottom-up) and corrective control (top-down) processes, suggesting differences in this circuitry amongst autistic compared to neurotypical children and adolescents. Sensorimotor control involves the processing of motion and position information in the primary visual cortex (V1), which projects to the SC to convert spatial data into motor commands and triggers brainstem burst neurons to generate catch-up saccades. The cerebellar vermis and posterior lobe then contribute to monitoring and refining eye velocity (de Brouwer et al., 2001; Quaia et al., 1999). Corrective control processes modulate catch-up saccade generation via the frontal eye fields (FEF) for error threshold monitoring, the posterior parietal cortex (PPC) for sensory integration, and the basal ganglia for timing modulation based on predicted motion patterns (Munoz & Wurtz, 1995). While sensorimotor processes of oculomotor functioning have been identified as compromised in autism, there have been inconclusive findings in ADHD (Castellanos et al., 2008; Castellanos et al., 2008; Suskauer et al., 2008). Meanwhile, corrective processing systems are historically implicated in ADHD, rather than autism (Chamorro et al., 2022; Maron et al., 2021). The current findings neither confirm nor refute whether sensorimotor control systems are intact in children and adolescents with ADHD, as no significant differences emerged between groups – possibly due to clinical variability overshadowing potential differences. However, the observed differences in performance in autistic and neurotypical groups, yet not ADHD, is consistent with literature pointing to motor impairment (including fine motor, gross motor, conscious fronto-striatal based movements and unconscious cerebellar-based movements) being predominantly driven by autistic traits (Rinehart & McGinley, 2010). These findings were not replicated in the Nottingham nor Kansas cohorts, which may have been underpowered due to their substantially smaller sample sizes, differences in testing stimuli, protocols and/or statistically significant age and sex difference between cohorts.

Previously, anti-saccade task performance has been frequently identified as a key difficulty in autism and ADHD. Specifically, individuals with autism-only and ADHD-only have been found to make significantly more directional errors during the AS task compared to neurotypical peers (Johnson et al., 2016; Maron et al., 2021). No statistically significant effects were elicited across any group performance on the AS task in the MAGNET Cohort, despite previously identified robust effects. This may be attributed to the substantially smaller sample size of participants who could (a) complete the task because they were at least 8 years old; and (b) accurately engage in the task. Supplementary Table F1 demonstrates 121 participants’ data were excluded from final analyses as they were unable to complete at least 6 viable trials. This is likely indicative that the AS task is perhaps too complex for children and adolescents with ADHD and autism to accurately complete, given it draws upon higher order cognitive functions including working memory, inhibitory control and language, in-line with previous findings of impaired performance on this task for both groups (Johnson et al., 2016; Maron et al., 2021). Supplementary Table F2 indicates younger children and adolescents had a significantly greater difficulty completing the AS task. Interestingly, a significant main effect for the number of directional errors was observed in the Kansas Cohort, such that autistic children and adolescents made significantly more directional errors than neurotypical children and adolescents. Notably, this pattern emerged despite the MAGNET Cohort task being comparatively more difficult given the narrow target amplitudes (i.e., 5° and 10°) compared to the Kansas Cohort (i.e., 12° and 24°). The lack of significant findings in the MAGNET Cohort may be attributed to reduced statistical power resulting from the separation of diagnostic groups. However, these findings were not replicated in the Nottingham Cohort, perhaps owing to smaller sample sizes of these cohorts, protocols, and/or significantly different age and sex differences between cohorts.

### Limitations

Several limitations of the present study warrant discussion. First, the broad age range of participants assumes stability in oculomotor outcomes across childhood to adolescence within diagnostic groups. Unfortunately, the uneven distribution of children and adolescents across age brackets precluded a detailed examination of oculomotor function and social communication differences within narrower age ranges, necessitating age as a covariate instead, which we regressed out as a nuisance variable. However, examining diagnostic group differences within age brackets has disadvantages, in that the age brackets would be arbitrary and could obscure differences in important developmental trends and lead to a loss of power. Future prospective cohort studies tracking changes in oculomotor function over time could offer a more nuanced understanding of cognitive differences between diagnostic groups throughout development by adopting multiple analytical approaches.

Second, some participants had additional co-occurring conditions including anxiety, which has demonstrated distinct patterns driven by a hypervigilance bias (Staab, 2014), potentially complicating oculomotor function. Nevertheless, including these children and adolescents enhances the sample’s representativeness of those with ADHD and autism, bolstering the ecological validity and generalizability of findings.

Third, a substantial number of participants were excluded from the analysis of the anti-saccade task to ensure data quality and reliable outcome measures, aligning with best practice recommendations (Fischer & Breitmeyer, 1987). However, this exclusion may have inadvertently omitted individuals with severe inhibition difficulties, potentially influencing the findings from the AS data.

Finally, the unequal group sizes in this study may be perceived as an additional limitation. While this doesn’t affect parameter estimates or effect sizes, it is argued to reduce power (Dibao-Dina et al., 2014). With this said, others suggest this is a false assumption, providing evidence that more power can be achieved when a larger control group is used (Oldfield, 2016). In any case, LME is regarded as a suitable approach for managing unbalanced data (Pinheiro, 2014).

## Conclusion

This paper identified distinct oculomotor markers capable of effectively discerning between children and adolescents with autism + ADHD or autism and neurotypical children and adolescents. Further research investigating potential differences between autism-only and ADHD-only versus autism + ADHD groups is warranted, although the findings may hold some promise for enhanced diagnostic efficiency and precision. Specifically, children and adolescents with autism + ADHD exhibited heightened variability in final eye positions, whilst autistic children and adolescents demonstrated significant inaccuracies during pursuit, when these groups were compared to a neurotypical group. These findings point to both sensorimotor control inefficiencies in children and adolescents with autism + ADHD, and both sensorimotor and corrective control inefficiencies in autistic children and adolescents compared to neurotypical children and adolescents. Future work should look towards characterizing the underlying neurobiology of differences in oculomotor functioning across the children and adolescents with autism, ADHD and autism + ADHD, and its links to symptomology.

## Supplementary Information

Below is the link to the electronic supplementary material.

Supplementary material 1 (DOCX 4,807 kb)

## Data Availability

Data is available upon request.
