# Supplementary material for: Oculomotor Function in Children and Adolescents with Autism, ADHD or Co-occurring Autism and ADHD
Source: J Autism Dev Disord. 2025 Jan 24;56(6):2391–407. doi: 10.1007/s10803-024-06718-3 (PMC13222194; doi:10.1007/s10803-024-06718-3)
Supplement: Supplementary file 1 — Supplementary material 1 (DOCX 4,807 kb) [file 10803_2024_6718_MOESM1_ESM.docx]

# Supplementary Material A

# Descriptive Oculomotor Statistics for MAGNET Cohort

## Supplementary Table A1. *Means and Standard Deviations for Transformed Oculomotor Measures Across Diagnostic Group*

| *M* (*SD*) | ADHD  *n* = 64 | | Autism  *n* = 66 | | ADHD+ autism  *n* = 146 | | Neurotypical  *n* = 129 |  |
| --- | --- | --- | --- | --- | --- | --- | --- | --- |
| **VGS Task** |  | |  | |  | |  |  |
| Relative time to peak velocity^a^ | 1.30 (0.27) | | 1.19 (0.34) | | 1.27 (0.27) | | 1.27 (0.26) |  |
| Gain^b^ | -0.02 (0.03) | | -0.02 (0.03) | | -0.03 (0.04) | | -0.02 (0.04) |  |
| Gain variability^b^ | -0.88 (0.18) | | -0.88 (0.22) | | -0.89 (0.2) | | -0.89 (0.22) |  |
| FEP^b^ | 0.00 (0.01) | | 0.00 (0.02) | | 0.00 (0.02) | | 0.00 (0.02) |  |
| FEP variability^b^ | -0.97 (0.2) | | -1.00 (0.23) | | -0.99 (0.21) | | -1.01 (0.22) |  |
| First Saccade Latency^c^ | 206.84 (34.15) | | 210.97 (36.85) | | 210.56 (38.87) | | 209.55 (38.15) |  |
| **Anti-saccade Task** |  | |  | |  | |  |  |
| Number of directional errors^b^ | 28.76 (5.60) | | 27.08 (5.04) | | 23.17 (7.81) | | 24.76 (8.10) |  |
| Number of anticipatory saccades^b^ | 1.62 (1.79) | | 1.08 (1.04) | | 2.06 (1.93) | | 1.71 (1.85) |  |
| **Sinusoidal Pursuit Task** | |  | |  | |  | | |
| Closed loop gain^a^ | 1.05 (1.42) | | 1.25 (0.86) | | 1.01 (1.55) | | 1.08 (1.40) |  |
| Number of catch-up saccades^b^ | 1.78 (0.12) | | 1.81 (0.14) | | 1.78 (0.18) | | 1.80 (0.15) |  |
| **Step-Ramp Pursuit Task** | |  | |  | |  | | |
| Open loop gain^b^ | -0.06 (0.13) | | -0.03 (0.12) | | -0.04 (0.14) | | -0.06 (0.14) |  |
| Number of catch-up saccades^b^ | 0.47 (0.08) | | 0.48 (0.11) | | 0.48 (0.10) | | 0.48 (0.10) |  |

*Note.* Means and standard deviations for transformed and imputed data. ADHD = Attention Deficit/Hyperactivity Disorder. Autism+ADHD = Co-occurring Attention Deficit/Hyperactivity Disorder and Autism. VGS = Visually Guided Saccade. FEP = Final Eye Position. ^a^Inverse transformation applied. ^b^Logarithmic transformation applied. ^c^Raw data, reported for descriptive purposes only – not included in main analyses. ^b^Only children aged eight years and over completed the anti-saccade task (*n* = 116).

# Supplementary Material B

## Completion Rates for Clinical Measures

## Supplementary Table B1. *Participant Completion Rates for Wechsler Scales, ADOS-2, CPRS, SRS, and Vineland*

| ADHD  *n =* 64 | Autism  *n =* 66 | ADHD+ autism  *n =* 146 | NT  *n =*  129 | Whole sample  *N =* 405 |
| --- | --- | --- | --- | --- |
| 54 | 57 | 137 | 96 | 344 |

*Note.* ADHD = Attention Deficit/Hyperactivity Disorder. Autism+ADHD = co-occurring Attention Deficit/Hyperactivity Disorder and Autism. NT = Neurotypical. Weschler scales = WISC-V, WAIS-IV or WASI-II. ADOS-2 = autism diagnostic observation schedule – second edition. CPRS = Conners’ parent rating scale. SRS = social responsiveness scale. Vineland = Vineland Adaptive Behavior Scale – 3^rd^ Edition. Motor = Vineland motor subscale.

**Supplementary Material C**

**Additional Procedural Details**

During three four-hour research visits to Monash University’s psychology clinics, children completed cognitive and neurocognitive assessments. Caregivers completed symptoms questionnaires online using Research Electronic Data Capture (REDCap; Harris et al., 2019; Harris et al., 2009). Caregivers were instructed to rate their child’s unmedicated ADHD and autism symptoms.

## Supplementary Material D

**Supplementary Table D.** *Checklist of minimal reporting for an eye tracking study (Dunn et al., 2023).*

| **Checklist Item** | **Details** |
| --- | --- |
| Manufacturer and model | SR Research Ltd. (all cohorts) |
| Software and firmware versions | Data Viewer Version 4.3.1 |
| Eye tracking technology | EyeLink 1000 (all cohorts) |
| Sampling frequency | 500 Hz (all cohorts) |
| Head movement restrictions | Headrest (MAGNET); Sticker used (Nottingham); Chinrest (Kansas) |
| Eye(s) recorded | Both eyes (all cohorts) |
| Parameters recorded | Gaze position data in screen pixel coordinates, with (0,0) at the top left of the display (default 1024x768 resolution) (all cohorts). |
| Environment lighting | MAGNET: Data was collected in a room with overhead fluorescent lighting and blackout blinds were drawn over a large window adjacent to the participant. Nottingham: Light levels were kept constant across participants, by using a light meter and keeping the lights low (but not until the environment was completely dark) via a dimmer. Kansas: Data was collected in darkened room with lights off. |
| Calibration | Saccade tasks: three-point; Pursuit tasks: five-point (MAGNET); nine-point (Nottingham); five-point (Kansas) |
| Measurement uncertainty | Accuracy: 0.25-0.5 degrees (all cohorts) |
| Data processing steps | Pre-processing using custom scripts developed by Langmead et al. (2024) in R and MATLAB (all cohorts) |
| Data loss | Refer to Supplementary Table E1 |
| Participant to display monitor distance | 84cm to monitor; 54cm to camera (MAGNET), 60cm (Nottingham), 61cm (Kansas) |

## Supplementary Material E

## Rates of Medication Use

## Supplementary Table E. *Medication Use by Diagnostic Group*

| Medication | ADHD | | Autism | | Autism+ADHD | Neurotypical | | |
| --- | --- | --- | --- | --- | --- | --- | --- | --- |
| **CNS Stimulants** | |  | |  |  | |  |  |
| Methylphenidate | | 20 (31.30%) | | 0 (0.00%) | 45 (30.80%) | | 1 (0.80%) |  |
| Dexamphetamine | | 1 (1.60%) | | 0 (0.00%) | 1 (0.70%) | | 0 (0.00%) |  |
| Lisdexamfetamine | | 5 (7.80%) | | 0 (0.00%) | 11 (7.50%) | | 0 (0.00%) |  |
| Armodafinil | | 0 (0.00%) | | 0 (0.00%) | 1 (0.70%) | | 0 (0.00%) |  |
| **Non-Stimulants** | |  | |  |  | |  |  |
| Atomoxetine | | 1 (1.60%) | | 0 (0.00%) | 2 (1.40%) | | 0 (0.00%) |  |
| Guanfacine | | 7 (10.90%) | | 0 (0.00%) | 16 (11.00%) | | 0 (0.00%) |  |
| Clonidine | | 1 (1.60%) | | 2 (3.00%) | 14 (9.60%) | | 0 (0.00%) |  |
| **Atypical Antipsychotics** | |  | |  |  | |  |  |
| Asenapine | | 0 (0.00%) | | 0 (0.00%) | 0 (0.00%) | | 0 (0.00%) |  |
| Clozapine | | 0 (0.00%) | | 0 (0.00%) | 0 (0.00%) | | 0 (0.00%) |  |
| Lurasidone | | 0 (0.00%) | | 0 (0.00%) | 1 (0.70%) | | 0 (0.00%) |  |
| Olanzapine | | 0 (0.00%) | | 0 (0.00%) | 0 (0.00%) | | 0 (0.00%) |  |
| Quetiapine | | 0 (0.00%) | | 0 (0.00%) | 2 (1.40%) | | 0 (0.00%) |  |
| Aripiprazole | | 1 (1.60%) | | 0 (0.00%) | 2 (1.40%) | | 0 (0.00%) |  |
| Risperidone | | 0 (0.00%) | | 1 (1.50%) | 4 (2.70%) | | 0 (0.00%) |  |
| Ziprasidone | | 0 (0.00%) | | 0 (0.00%) | 0 (0.00%) | | 0 (0.00%) |  |
| **Antidepressants** | |  | |  |  | |  |  |
| Mirtazapine | | 0 (0.00%) | | 0 (0.00%) | 0 (0.00%) | | 0 (0.00%) |  |
| Dosulepin | | 0 (0.00%) | | 0 (0.00%) | 0 (0.00%) | | 0 (0.00%) |  |
| Citalopram | | 0 (0.00%) | | 0 (0.00%) | 0 (0.00%) | | 0 (0.00%) |  |
| Escitalopram | | 0 (0.00%) | | 0 (0.00%) | 0 (0.00%) | | 0 (0.00%) |  |
| Fluvoxamine | | 2 (3.10%) | | 1 (1.50%) | 11 (7.50%) | | 2 (1.60%) |  |
| Fluoxetine | | 5 (7.80%) | | 3 (4.50%) | 18 (12.30%) | | 2 (1.60%) |  |
| Sertraline | | 1 (1.60%) | | 1 (1.50%) | 2 (1.40%) | | 0 (0.00%) |  |
| Paroxetine | | 0 (0.00%) | | 0 (0.00%) | 0 (0.00%) | | 0 (0.00%) |  |
| Venlafaxine | | 0 (0.00%) | | 0 (0.00%) | 0 (0.00%) | | 0 (0.00%) |  |
| Desvenlafaxine | | 0 (0.00%) | | 0 (0.00%) | 1 (0.70%) | | 0 (0.00%) |  |
| **Anticonvulsants** | |  | |  |  | |  |  |
| Gabapentin | | 1 (1.60%) | | 0 (0.00%) | 0 (0.00%) | | 0 (0.00%) |  |
| **Hormones** | |  | |  |  | |  |  |
| Testosterone | | 0 (0.00%) | | 0 (0.00%) | 0 (0.00%) | | 1 (0.80%) |  |
| Melatonin | | 11 (17.20%) | | 6 (9.10%) | 37 (25.30%) | | 6 (4.70%) |  |

*Note.* ADHD = attention deficit/hyperactivity disorder. Autism = autism spectrum disorder. Autism+ADHD = co-occurring attention deficit/hyperactivity disorder and autism spectrum disorder. CNS = Central Nervous System. Medication categories are not mutually exclusive, the same children may be counted for more than one. Percentages are column percentages.

**Supplementary Material F**

**Supplementary** **Table F1.** *Reasons for participant non-completion or data removal from anti-saccade task for participants over 8 years.*

| *Exclusion reason* | ADHD | Autism | ADHD+ autism | NT | Whole sample |
| --- | --- | --- | --- | --- | --- |
| Less than 6 valid trials | 22 | 17 | 51 | 31 | 121 |
| Child didn't understand instructions | - | 2 | - | - | 2 |
| Task not attempted for behavioral reasons | - | 1 | 3 | 1 | 5 |
| Task not attempted: no reason provided | - | 2 | - | 1 | 3 |
| Technical error | - | 2 | 3 | 4 | 9 |

*Note.* ADHD = attention deficit/hyperactivity disorder. Autism+ADHD = co-occurring attention deficit/hyperactivity disorder and autism spectrum disorder. NT = Neurotypical. Dash indicates no participants eliminated for this reason.

**Supplementary** **Table F2.** *Partial Correlational Investigations of Anti-saccade Non-completion Reasons Controlling for Diagnostic Group*

| *Clinical Measure* | Task not attempted or less than 6 valid trials | |
| --- | --- | --- |
|  | *r* | *p* |
| Sex | -0.34 | .505 |
| **Age (years)** | **0.208** | **<.001** |
| CPRS Inattentive | .092 | .069 |
| CPRS Hyperactive | .054 | .281 |

*Note.* r = Pearson’s correlation coefficient. CPRS = Conners’ Parent Rating Scale. **Bold** = significant correlation at Bonferroni-corrected *p* < .004.

**Supplementary** **Table F3.** *Logistic Regression of Diagnostic Group and Age by Ability to Complete Anti-Saccade Task*

|  | B | SE | Wald | *df* | *p* | Exp(B) |
| --- | --- | --- | --- | --- | --- | --- |
|  |  |  |  |  |  |  |
| **Diagnostic Group** | **1.451** | **0.617** | **5.525** | **1** | **0.019** | **4.266** |
| Age (years) | -0.136 | 0.097 | 1.950 | 1 | 0.163 | 0.873 |
| **Diagnostic Group*Age** | **-0.120** | **0.054** | **4.854** | **1** | **0.028** | **0.887** |
| Constant | 1.476 | 1.131 | 1.703 | 1 | 0.192 | 4.375 |

*Note. B = B coefficient,* SE = Standard Error, Wald = Wald Chi-Square value, df = Degrees of Freedom, p = Probability, Exp(B) = Exponentiation of the B coefficient. **Bold** = significant at *p* <.05. Hosmer and Lemeshow Test: χ2(8)=11.40, *p* =.180. The -2 Log Likelihood value for the model was 283.83. The Cox & Snell R Square value was 0.142, and the Nagelkerke R Square value was 0.190, indicating that the model explained between 13.2% and 19.0% of the variance in the outcome variable.

**Supplementary Material G**

## Linear Mixed Effects Model Intraclass Correlation and Fit Statistics

## Supplementary Table G1. *Linear Mixed Effects Model Fit Statistics for Models Examining Diagnostic Group and Sex for Each Oculomotor Outcome*

|  | ICC | -2LL | AIC | BIC |
| --- | --- | --- | --- | --- |
| **VGS Task** |  |  |  |  |
| Relative time to peak velocity^a^ | 0.33 | -1463.94 | -1459.94 | -1451.96 |
| Gain^b^ | 0.33 | -2810.61 | -2806.61 | -2798.63 |
| Gain variability^b^ | 0.33 | -1207.82 | -1203.82 | -1195.84 |
| FEP^b^ | 0.33 | -3159.80 | -3155.80 | -3147.82 |
| FEP variability^b^ | 0.33 | -940.36 | -936.36 | -928.38 |
| **Anti-saccade Task** |  |  |  |  |
| Number of directional errors^b^ | 0.33 | 516.96 | 520.96 | 526.38 |
| Number of anticipatory saccades^b^ | 0.33 | 148.97 | 152.97 | 158.39 |
| **Sinusoidal Pursuit Task** |  |  |  |  |
| Closed loop gain^a^ | 0.33 | 532.74 | 536.74 | 544.73 |
| Number of catch-up saccades^b^ | 0.33 | -2081.03 | -2077.03 | -2069.05 |
| **Step-Ramp Pursuit Task** |  |  |  |  |
| Open loop gain^b^ | 0.33 | 532.74 | 536.74 | 544.73 |
| Number of catch-up saccades^b^ | 0.33 | 2116.35 | 2120.35 | 2128.33 |

*Note.* ICC = Adjusted Intra-Class Correlation. -2LL = -2 Restricted Log Likelihood. AIC = Akaike's Information Criterion. BIC = Schwarz's Bayesian Information Criterion. VGS = Visually Guided Saccade. FEP = Final Eye Position. ^a^Inverse transformation. ^b^Log_10_ transformation.

**Supplementary Material H**

**Data Transformations**

All oculomotor outcomes demonstrated significant skewness and were transformed prior to MI.

Log_10,_ square root, inverse and arcsine transformations were trialed where applicable, and Q-Q plots were examined for normality. Log_10_ transformations produced the most parsimonious outcomes for gain, gain variability, FEP, FEP variability, anti-saccade outcomes, number of catch-up saccades in the smooth pursuit task and step-ramp outcomes, whilst inverse transformations produced the most parsimonious outcomes for the Relative time to peak velocity and open loop gain.

# Supplementary Material I

## Linear Mixed Effects Model Main Effect Statistics

## Supplementary Table I. *Linear Mixed Effects Model Fixed Effects Statistics for Main Effects of Diagnostic Group and a Regression Residual Controlling for Sex and Age*

| Oculomotor Measures | | *df_Num_* | *df_Den_* | *F* | *d* | *p* |
| --- | --- | --- | --- | --- | --- | --- |
| **VGS Task** |  |  |  |  |  |  |
| Relative time to peak velocity | |  |  |  |  |  |
|  | Diagnostic Group | 3 | 400 | 1.74 | 0.227 | .249 |
|  | Intercept | 1 | 400 | 2367.35 |  | <.001 |
|  | Regression Residual | 1 | 400 | 29203.60 |  | <.001 |
| Gain |  |  |  |  |  |  |
|  | Diagnostic Group | 3 | 400 | 0.73 | 0.147 | .576 |
|  | Intercept | 1 | 400 | 16.16 |  | .058 |
|  | Regression Residual | 1 | 400 | 16564.82 |  | <.001 |
| Gain variability | |  |  |  |  |  |
|  | Diagnostic Group | 3 | 400 | 2.50 | 0.272 | .084 |
|  | Intercept | 1 | 400 | 568.01 |  | <.001 |
|  | Regression Residual | 1 | 400 | 7804.88 |  | <.001 |
| FEP |  |  |  |  |  |  |
|  | Diagnostic Group | 3 | 400 | 0.92 | 0.165 | .457 |
|  | Intercept | 1 | 400 | 0.02 |  | .918 |
|  | Regression Residual | 1 | 400 | 11541.27 |  | <.001 |
| FEP variability | |  |  |  |  |  |
|  | **Diagnostic Group** | **3** | **400** | **3.38** | **0.316** | **.025** |
|  | Intercept | 1 | 400 | 377.49 |  | <.001 |
|  | Regression Residual | 1 | 400 | 3410.09 |  | <.001 |
| **Anti-saccade Task**    Number of directional errors | |  |  |  |  |  |
|  |  |  |  |  |  |  |
|  | Diagnostic Group | 3 | 111 | 0.36 | 0.194 | .784 |
|  | Intercept | 1 | 111 | 239.42 |  | <.001 |
|  | Regression Residual | 1 | 111 | 1219.74 |  | <.001 |
| Number of anticipatory saccades | |  |  |  |  |  |
|  | Diagnostic Group | 3 | 111 | 0.33 | 0.186 | .804 |
|  | Intercept | 1 | 111 | 31.70 |  | <.001 |
|  | Regression Residual | 1 | 111 | 2207.27 |  | <.001 |
| **Sinusoidal Pursuit Task**    Closed loop gain | |  |  |  |  |  |
|  |  |  |  |  |  |  |
|  | Diagnostic Group | 3 | 400 | 2.94 | 0.295 | .092 |
|  | Intercept | 1 | 400 | 17.72 |  | <.001 |
|  | Regression Residual | 1 | 400 | 38163.02 |  | <.001 |
| Number of catchup saccades | |  |  |  |  |  |
|  | Diagnostic Group | 3 | 400 | 3.55 | 0.324 | .034 |
|  | Intercept | 1 | 400 | 22732.84 |  | <.001 |
|  | Regression Residual | 1 | 400 | 39006.54 |  | <.001 |
| **Step-Ramp Pursuit Task**    Open loop gain | |  |  |  |  |  |
|  |  |  |  |  |  |  |
|  | Diagnostic Group | 3 | 400 | 2.94 | 0.295 | .092 |
|  | Intercept | 1 | 400 | 17.72 |  | <.001 |
|  | Regression Residual | 1 | 400 | 38163.02 |  | <.001 |
| Number of catchup saccades | |  |  |  |  |  |
|  | **Diagnostic Group** | **3** | **400** | **3.09** | **0.302** | **.027** |
|  | Intercept | 1 | 400 | 0.96 |  | .529 |
|  | Regression Residual | 1 | 400 | 43698.62 |  | <.001 |

*Note. df_Num_* = numerator degrees of freedom. *df_Den_* = denominator degrees of freedom. *F* = linear mixed effects model *F*-statistic. *d* = Cohen’s *d*. *p* = *p*-value. VGS = visually guided saccade. FEP = final eye position. **Bold** = significant main effect. The Benjamini Hochberg corrected alpha value for VGS FEP variability is *p* < .030; Step-Ramp Number of catchup saccades is *p* < .031 and Sinusoidal Pursuit Number of catchup saccades is *p* < .033.

# Supplementary Material J

## Linear Mixed Effects Models Pairwise Comparisons

## Supplementary Table J. *Linear Mixed Effects Model Diagnostic Group Pairwise Comparisons*

|  | | |  | |  |  |  |  | 95% CI | |
| --- | --- | --- | --- | --- | --- | --- | --- | --- | --- | --- |
| Oculomotor Measures | | | Mean Diff. | | Std. Error | *df* | *d* | *p* | Lower | Upper |
| **Visually Guided Saccade** | | |  | |  |  |  |  |  |  |
| Gain | | |  | |  |  |  |  |  |  |
| NT | Autism |  | | 0.008 | 0.006 | 400 | 0.026 | 0.797 | -0.008 | 0.023 |
|  | ADHD |  | | 0.001 | 0.006 | 400 | 0.002 | 1.000 | -0.015 | 0.016 |
|  | Autism+ADHD |  | | 0.007 | 0.005 | 400 | 0.023 | 0.503 | -0.004 | 0.020 |
| Autism | NT |  | | -0.008 | 0.006 | 400 | -0.026 | 0.797 | -0.023 | 0.008 |
|  | ADHD |  | | -0.007 | 0.007 | 400 | -0.031 | 1.000 | -0.025 | 0.011 |
|  | Autism+ADHD |  | | 0.001 | 0.006 | 400 | 0.002 | 1.000 | -0.014 | 0.016 |
| ADHD | NT |  | | -0.001 | 0.006 | 400 | -0.002 | 1.000 | -0.016 | 0.015 |
|  | Autism |  | | 0.007 | 0.007 | 400 | 0.031 | 1.000 | -0.011 | 0.025 |
|  | Autism+ADHD |  | | 0.008 | 0.006 | 400 | 0.025 | 0.847 | -0.007 | 0.023 |
| Autism+ADHD | NT |  | | -0.007 | 0.005 | 400 | -0.023 | 0.503 | -0.020 | 0.004 |
|  | Autism |  | | -0.001 | 0.006 | 400 | -0.002 | 1.000 | -0.016 | 0.014 |
|  | ADHD |  | | -0.008 | 0.006 | 400 | -0.025 | 0.847 | -0.023 | 0.007 |
| Gain | | |  | |  |  |  |  |  |  |
| NT | Autism |  | | -0.001 | 0.001 | 400 | -0.020 | 0.930 | -0.004 | 0.002 |
|  | ADHD |  | | 0.000 | 0.001 | 400 | -0.003 | 1.000 | -0.003 | 0.003 |
|  | Autism+ADHD |  | | -0.001 | 0.001 | 400 | -0.014 | 0.866 | -0.003 | 0.001 |
| Autism | NT |  | | 0.001 | 0.001 | 400 | 0.020 | 0.930 | -0.002 | 0.004 |
|  | ADHD |  | | 0.001 | 0.001 | 400 | 0.022 | 0.999 | -0.002 | 0.004 |
|  | Autism+ADHD |  | | 0.000 | 0.001 | 400 | 0.004 | 1.000 | -0.003 | 0.003 |
| ADHD | NT |  | | 0.000 | 0.001 | 400 | 0.003 | 1.000 | -0.003 | 0.003 |
|  | Autism |  | | -0.001 | 0.001 | 400 | -0.022 | 0.999 | -0.004 | 0.002 |
|  | Autism+ADHD |  | | -0.001 | 0.001 | 400 | -0.012 | 1.000 | -0.004 | 0.002 |
| Autism+ADHD | NT |  | | 0.001 | 0.001 | 400 | 0.014 | 0.866 | -0.001 | 0.003 |
|  | Autism |  | | 0.000 | 0.001 | 400 | -0.004 | 1.000 | -0.003 | 0.003 |
|  | ADHD |  | | 0.001 | 0.001 | 400 | 0.012 | 1.000 | -0.002 | 0.004 |
| Gain variability | | |  | |  |  |  |  |  |  |
| NT | Autism |  | | -0.015 | 0.008 | 400 | -0.039 | 0.386 | -0.036 | 0.006 |
|  | ADHD |  | | 0.000 | 0.008 | 400 | 0.000 | 1.000 | -0.021 | 0.021 |
|  | Autism+ADHD |  | | -0.012 | 0.006 | 400 | -0.027 | 0.344 | -0.030 | 0.004 |
| Autism | NT |  | | 0.015 | 0.008 | 400 | 0.039 | 0.386 | -0.006 | 0.036 |
|  | ADHD |  | | 0.015 | 0.009 | 400 | 0.051 | 0.572 | -0.009 | 0.040 |
|  | Autism+ADHD |  | | 0.002 | 0.008 | 400 | 0.006 | 1.000 | -0.018 | 0.023 |
| ADHD | NT |  | | 0.000 | 0.008 | 400 | 0.000 | 1.000 | -0.021 | 0.021 |
|  | Autism |  | | -0.015 | 0.009 | 400 | -0.051 | 0.572 | -0.040 | 0.009 |
|  | Autism+ADHD |  | | -0.013 | 0.008 | 400 | -0.031 | 0.601 | -0.034 | 0.008 |
| Autism+ADHD | NT |  | | 0.012 | 0.006 | 400 | 0.027 | 0.344 | -0.004 | 0.030 |
|  | Autism |  | | -0.002 | 0.008 | 400 | -0.006 | 1.000 | -0.023 | 0.018 |
|  | ADHD |  | | 0.013 | 0.008 | 400 | 0.032 | 0.601 | -0.008 | 0.034 |
| FEP | | |  | |  |  |  |  |  |  |
| NT | Autism |  | | 0.000 | 0.001 | 400 | 0.010 | 1.000 | -0.002 | 0.002 |
|  | ADHD |  | | -0.001 | 0.001 | 400 | -0.018 | 0.986 | -0.003 | 0.001 |
|  | Autism+ADHD |  | | -0.001 | 0.001 | 400 | -0.014 | 0.933 | -0.002 | 0.001 |
| Autism | NT |  | | 0.000 | 0.001 | 400 | -0.009 | 1.000 | -0.002 | 0.002 |
|  | ADHD |  | | -0.001 | 0.001 | 400 | -0.028 | 0.960 | -0.003 | 0.001 |
|  | Autism+ADHD |  | | -0.001 | 0.001 | 400 | -0.031 | 0.898 | -0.003 | 0.001 |
| ADHD | NT |  | | 0.001 | 0.001 | 400 | 0.017 | 0.986 | -0.001 | 0.003 |
|  | Autism |  | | 0.001 | 0.001 | 400 | 0.029 | 0.960 | -0.001 | 0.003 |
|  | Autism+ADHD |  | | 0.000 | 0.001 | 400 | 0.005 | 1.000 | -0.002 | 0.002 |
| Autism+ADHD | NT |  | | 0.001 | 0.001 | 400 | 0.014 | 0.933 | -0.001 | 0.002 |
|  | Autism |  | | 0.001 | 0.001 | 400 | 0.022 | 0.898 | -0.001 | 0.003 |
|  | ADHD |  | | 0.000 | 0.001 | 400 | -0.004 | 1.000 | -0.002 | 0.002 |
| FEP variability | | |  | |  |  |  |  |  |  |
| NT | Autism |  | | -0.017 | 0.011 | 400 | -0.006 | 0.716 | -0.046 | 0.012 |
|  | ADHD |  | | -0.003 | 0.011 | 400 | -0.040 | 1.000 | -0.033 | 0.027 |
|  | Autism+ADHD |  | | **-0.022** | **0.009** | **400** | **0.028** | **0.035** | **-0.049** | **-0.002** |
| Autism | NT |  | | 0.017 | 0.011 | 400 | 0.026 | 0.716 | -0.012 | 0.046 |
|  | ADHD |  | | 0.014 | 0.013 | 400 | -0.021 | 1.000 | -0.020 | 0.048 |
|  | Autism+ADHD |  | | -0.009 | 0.011 | 400 | 0.005 | 1.000 | -0.037 | 0.020 |
| ADHD | NT |  | | 0.003 | 0.011 | 400 | -0.026 | 1.000 | -0.027 | 0.033 |
|  | Autism |  | | -0.014 | 0.013 | 400 | -0.054 | 1.000 | -0.048 | 0.020 |
|  | Autism+ADHD |  | | -0.023 | 0.011 | 400 | 0.038 | 0.238 | -0.052 | 0.006 |
| Autism+ADHD | NT |  | | **0.022** | **0.009** | **400** | **0.014** | **0.035** | **0.002** | **0.049** |
|  | Autism |  | | 0.009 | 0.011 | 400 | 0.039 | 1.000 | -0.020 | 0.037 |
|  | ADHD |  | | 0.023 | 0.011 | 400 | 0.000 | 0.238 | -0.006 | 0.052 |
| **Anti-saccade Task** | | |  | |  |  |  |  |  |  |
| Number of directional errors | | |  | |  |  |  |  |  |  |
| NT | Autism |  | | 0.009 | 0.693 | 111 | 0.030 | 1.000 | -1.853 | 1.871 |
|  | ADHD |  | | 0.281 | 0.597 | 111 | -0.035 | 1.000 | -1.322 | 1.884 |
|  | Autism+ADHD |  | | -0.330 | 0.516 | 111 | -0.001 | 1.000 | -1.717 | 1.057 |
| Autism | NT |  | | -0.009 | 0.693 | 111 | 0.029 | 1.000 | -1.871 | 1.853 |
|  | ADHD |  | | 0.272 | 0.736 | 111 | -0.044 | 1.000 | -1.705 | 2.249 |
|  | Autism+ADHD |  | | -0.339 | 0.699 | 111 | -0.031 | 1.000 | -2.218 | 1.540 |
| ADHD | NT |  | | -0.281 | 0.597 | 111 | -0.029 | 1.000 | -1.884 | 1.322 |
|  | Autism |  | | -0.272 | 0.736 | 111 | -0.079 | 1.000 | -2.249 | 1.705 |
|  | Autism+ADHD |  | | -0.611 | 0.604 | 111 | 0.035 | 1.000 | -2.235 | 1.012 |
| Autism+ADHD | NT |  | | 0.330 | 0.516 | 111 | 0.034 | 1.000 | -1.057 | 1.717 |
|  | Autism |  | | 0.339 | 0.699 | 111 | 0.067 | 1.000 | -1.540 | 2.218 |
|  | ADHD |  | | 0.611 | 0.604 | 111 | 0.000 | 1.000 | -1.012 | 2.235 |
| Number of anticipatory saccades | | |  | |  |  |  |  |  |  |
| NT | Autism |  | | 0.125 | 0.132 | 111 | 0.029 | 1.000 | -0.230 | 0.480 |
|  | ADHD |  | | 0.052 | 0.111 | 111 | 0.033 | 1.000 | -0.246 | 0.351 |
|  | Autism+ADHD |  | | 0.061 | 0.100 | 111 | -0.064 | 1.000 | -0.207 | 0.329 |
| Autism | NT |  | | -0.125 | 0.132 | 111 | -0.040 | 1.000 | -0.480 | 0.230 |
|  | ADHD |  | | -0.073 | 0.142 | 111 | -0.043 | 1.000 | -0.454 | 0.308 |
|  | Autism+ADHD |  | | -0.064 | 0.134 | 111 | -0.030 | 1.000 | -0.423 | 0.295 |
| ADHD | NT |  | | -0.052 | 0.111 | 111 | 0.040 | 1.000 | -0.351 | 0.246 |
|  | Autism |  | | 0.073 | 0.142 | 111 | 0.006 | 1.000 | -0.308 | 0.454 |
|  | Autism+ADHD |  | | 0.009 | 0.113 | 111 | -0.034 | 1.000 | -0.294 | 0.312 |
| Autism+ADHD | NT |  | | -0.061 | 0.100 | 111 | 0.033 | 1.000 | -0.329 | 0.207 |
|  | Autism |  | | 0.064 | 0.134 | 111 | -0.005 | 1.000 | -0.295 | 0.423 |
|  | ADHD |  | | -0.009 | 0.113 | 111 | 0.000 | 1.000 | -0.312 | 0.294 |
| **Sinusoidal Pursuit Task** | | |  | |  |  |  |  |  |  |
| Closed loop gain | | |  | |  |  |  |  |  |  |
| NT | Autism |  | | -0.127 | 0.070 | 400 | -0.039 | 0.413 | -0.319 | 0.050 |
|  | ADHD |  | | -0.139 | 0.070 | 400 | -0.023 | 0.346 | -0.326 | 0.047 |
|  | Autism+ADHD |  | | -0.081 | 0.056 | 400 | 0.032 | 0.335 | -0.278 | 0.017 |
| Autism | NT |  | | 0.127 | 0.070 | 400 | -0.001 | 0.413 | -0.050 | 0.319 |
|  | ADHD |  | | -0.005 | 0.081 | 400 | 0.001 | 1.000 | -0.219 | 0.209 |
|  | Autism+ADHD |  | | 0.004 | 0.068 | 400 | 0.037 | 0.953 | -0.177 | 0.185 |
| ADHD | NT |  | | 0.139 | 0.070 | 400 | 0.001 | 0.346 | -0.047 | 0.326 |
|  | Autism |  | | 0.005 | 0.081 | 400 | 0.003 | 1.000 | -0.209 | 0.219 |
|  | Autism+ADHD |  | | 0.009 | 0.069 | 400 | 0.022 | 1.000 | -0.174 | 0.192 |
| Autism+ADHD | NT |  | | 0.081 | 0.056 | 400 | -0.001 | 0.335 | -0.017 | 0.278 |
|  | Autism |  | | -0.004 | 0.068 | 400 | -0.002 | 0.953 | -0.185 | 0.177 |
|  | ADHD |  | | -0.009 | 0.069 | 400 | 0.000 | 1.000 | -0.192 | 0.174 |
| Number of catchup saccades | | |  | |  |  |  |  |  |  |
| NT | Autism |  | | 0.005 | 0.003 | 400 | 0.033 | 0.531 | -0.003 | 0.012 |
|  | ADHD |  | | 0.006 | 0.003 | 400 | -0.033 | 0.268 | -0.002 | 0.013 |
|  | Autism+ADHD |  | | 0.005 | 0.002 | 400 | 0.006 | 0.066 | 0.001 | 0.012 |
| Autism | NT |  | | -0.005 | 0.003 | 400 | 0.015 | 0.531 | -0.012 | 0.003 |
|  | ADHD |  | | 0.001 | 0.003 | 400 | -0.052 | 1.000 | -0.007 | 0.009 |
|  | Autism+ADHD |  | | 0.002 | 0.003 | 400 | -0.007 | 0.926 | -0.005 | 0.009 |
| ADHD | NT |  | | -0.006 | 0.003 | 400 | 0.007 | 0.268 | -0.013 | 0.002 |
|  | Autism |  | | -0.001 | 0.003 | 400 | -0.043 | 1.000 | -0.009 | 0.007 |
|  | Autism+ADHD |  | | 0.001 | 0.003 | 400 | -0.014 | 1.000 | -0.006 | 0.008 |
| Autism+ADHD | NT |  | | -0.005 | 0.002 | 400 | -0.007 | 0.066 | -0.012 | -0.001 |
|  | Autism |  | | -0.002 | 0.003 | 400 | 0.000 | 0.926 | -0.009 | 0.005 |
|  | ADHD |  | | -0.001 | 0.003 | 400 | 0.000 | 1.000 | -0.008 | 0.006 |
| **Step-Ramp Pursuit Task** | | |  | |  |  |  |  |  |  |
| Open loop gain | | |  | |  |  |  |  |  |  |
| NT | Autism |  | | -0.127 | 0.070 | 400 | -0.007 | 0.413 | -0.319 | 0.050 |
|  | ADHD |  | | -0.139 | 0.070 | 400 | 0.000 | 0.346 | -0.326 | 0.047 |
|  | Autism+ADHD |  | | -0.081 | 0.056 | 400 | 0.000 | 0.335 | -0.278 | 0.017 |
| Autism | NT |  | | 0.127 | 0.070 | 400 | -0.007 | 0.413 | -0.050 | 0.319 |
|  | ADHD |  | | -0.005 | 0.081 | 400 | 0.000 | 1.000 | -0.219 | 0.209 |
|  | Autism+ADHD |  | | 0.004 | 0.068 | 400 | 0.000 | 0.953 | -0.177 | 0.185 |
| ADHD | NT |  | | 0.139 | 0.070 | 400 | -0.007 | 0.346 | -0.047 | 0.326 |
|  | Autism |  | | 0.005 | 0.081 | 400 | 0.000 | 1.000 | -0.209 | 0.219 |
|  | Autism+ADHD |  | | 0.009 | 0.069 | 400 | 0.000 | 1.000 | -0.174 | 0.192 |
| Autism+ADHD | NT |  | | 0.081 | 0.056 | 400 | -0.007 | 0.335 | -0.017 | 0.278 |
|  | Autism |  | | -0.004 | 0.068 | 400 | 0.000 | 0.953 | -0.185 | 0.177 |
|  | ADHD |  | | -0.009 | 0.069 | 400 | 0.000 | 1.000 | -0.192 | 0.174 |
| Number of catchup saccades | | |  | |  |  |  |  |  |  |
| NT | Autism |  | | **-0.084** | **0.636** | **400** | **0.002** | **0.015** | **-1.771** | **1.602** |
|  | ADHD |  | | -0.025 | 0.643 | 400 | 0.001 | 1.000 | -1.730 | 1.679 |
|  | Autism+ADHD |  | | -0.034 | 0.508 | 400 | 0.001 | 0.912 | -1.382 | 1.314 |
| Autism | NT |  | | **0.084** | **0.636** | **400** | **0.001** | **0.015** | **-1.602** | **1.771** |
|  | ADHD |  | | 0.059 | 0.737 | 400 | -0.002 | 0.419 | -1.894 | 2.012 |
|  | Autism+ADHD |  | | 0.050 | 0.623 | 400 | 0.000 | 0.326 | -1.601 | 1.702 |
| ADHD | NT |  | | 0.025 | 0.643 | 400 | 0.001 | 1.000 | -1.679 | 1.730 |
|  | Autism |  | | -0.059 | 0.737 | 400 | -0.002 | 0.419 | -2.012 | 1.894 |
|  | Autism+ADHD |  | | -0.009 | 0.630 | 400 | 0.000 | 1.000 | -1.678 | 1.661 |
| Autism+ADHD | NT |  | | 0.034 | 0.508 | 400 | 0.000 | 0.912 | -1.314 | 1.382 |
| NT | Autism |  | | -0.050 | 0.623 | 400 | 0.000 | 0.326 | -1.702 | 1.601 |
|  | ADHD |  | | 0.009 | 0.630 | 400 | 0.000 | 1.000 | -1.661 | 1.678 |

*Note.* Mean diff = Mean Difference. Std. Error = Standard Error. *df* = Degrees of freedom. *d* = Cohen’s *d*. *p* = *p*-value. 95% CI = 95% Confidence Interval. Lower = Lower Bound. Upper = Upper Bound. NT = Neurotypical. Autism = Autism Spectrum Disorder. ADHD = Attention-Deficit/Hyperactivity Disorder. Autism+ADHD = Co-Occurring ADHD And Autism. FEP = Final Eye Position. **Bold** = statistically significant after Bonferroni correction. Significance has only been interpreted following a significant Main Effect.

**Supplementary Material K**

**Nottingham Cohort Confirmatory Dataset**

**Supplementary** **Table K1.** *Nottingham Cohort: Sample characteristics for ADHD, autism, Autism+ADHD and Neurotypical groups.*

|  | ADHD | Autism | ADHD+ autism | NT | Whole sample |
| --- | --- | --- | --- | --- | --- |
|  | *n =* 22 | *n =* 17 | *n =* 32 | *n =* 30 | *N =* 101 |
| Child age (years) | 10.48 (2.16) | 11.00 (2.11) | 10.84 (1.53) | 10.90 (2.44) | 10.78 (2.04) |
| Child sex |  |  |  |  |  |
| Male | 14 | 10 | 24 | 17 | 65 |
| Female | 8 | 7 | 8 | 13 | 36 |
| FSIQ-4 | 109.05 (11.75) | 103.53 (15.41) | 102.06 (19.29) | 116.53 (12.28) | 108.13 (16.43) |
| ADOS-2^a^ | 4.68 (3.63) | 14.00 (4.86) | 14.03 (4.98) | NA | 15.10 (9.40) |
| SCQ^b^ | 15.64 (6.75) | 19.47 (5.60) | 21.16 (6.23) | 5.11 (7.78) | 18.72 (6.59) |

*Note.* Values = Mean (SD). ADHD = Attention Deficit/Hyperactivity Disorder. Autism = Autism Spectrum Disorder. Autism+ADHD = Co-Occurring Attention Deficit/Hyperactivity Disorder And Autism Spectrum Disorder. NT = Neurotypical. Child sex = frequency (percentage). FSIQ-4 = Full Scale Intelligence Quotient. ADOS-2 = Autism Diagnostic Observation Schedule – Second Edition Social Affect + Restrictive And Repetitive Behavior Total Score. SCQ = Social Communication Questionnaire Total Score. NA = Not Applicable as no data were collected for this group. ^a^*n* = 67. ^b^*n* = 99.

## Supplementary Table K2. *Nottingham Cohort: Means and Standard Deviations for the Oculomotor Function Outcomes Across Diagnostic Group and for Males and Females*

| *M (SD)* | ADHD  *n =* 22 | Autism  *n =* 17 | ADHD+ autism  *n =* 32 | Neurotypical  *n =* 30 |
| --- | --- | --- | --- | --- |
| Relative time to peak velocity | 0.84 (0.10) | 0.93 (0.34) | 0.84 (0.06) | 0.85 (0.09) |
| Gain | 0.92 (0.06) | 0.93 (0.06) | 0.92 (0.06) | 0.92 (0.05) |
| Gain variability | 0.13 (0.05) | 0.15 (0.07) | 0.12 (0.04) | 0.12 (0.05) |
| FEP | 1.01 (0.03) | 1 (0.04) | 0.99 (0.03) | 0.99 (0.05) |
| FEP variability | 0.09 (0.04) | 0.1 (0.05) | 0.09 (0.04) | 0.09 (0.04) |
| First saccade latency^a^ | 267.96 (100.99) | 233.08 (48.23) | 251.65 (76.35) | 269.29 (80.21) |

*Note.* ADHD = Attention Deficit/Hyperactivity Disorder. Autism+ADHD = Co-occurring Attention Deficit/Hyperactivity Disorder and Autism. FEP = Final Eye Position. ^a^Reported for descriptive purposes only – not included in main analyses.

**Supplementary** **Table K3.** *Nottingham Cohort: Medication Use by Diagnostic Group*

| Medication | ADHD | | Autism | | Autism+ADHD | NT | | |
| --- | --- | --- | --- | --- | --- | --- | --- | --- |
| Methylphenidate | | 4 (18.18%) | | 0 (0.00%) | 2 (0.06%) | | 0 (0.00%) |  |
| Sertraline | | 0 (0.00%) | | 0 (0.00%) | 1 (0.03%) | | 0 (0.00%) |  |
| Melatonin | | 1 (0.05%) | | 0 (0.00%) | 2 (0.06%) | | 0 (0.00%) |  |

*Note.* 75% of respondents did not report whether or not medication was used. ADHD = Attention Deficit/Hyperactivity Disorder. Autism = Autism Spectrum Disorder. Autism+ADHD = Co-Occurring Attention Deficit/Hyperactivity Disorder and Autism Spectrum Disorder. NT = Neurotypical. Medication categories are not mutually exclusive, the same children may be counted for more than one. Percentages are column percentages.

## Supplementary Table K4. *Nottingham Cohort: Linear Mixed Effects Model Fixed Effects Statistics for Diagnostic Group, Age and Sex*

| Oculomotor Measures | | *df_Num_* | *df_Den_* | *F* | *d* | *p* |
| --- | --- | --- | --- | --- | --- | --- |
| Relative time to peak velocity | |  |  |  |  |  |
|  | Intercept | 1 | 96 | 41536.13 |  | <.001 |
|  | Diagnostic Group | 3 | 96 | 2.34 | 0.334 | .078 |
|  | Regression Residual | 1 | 96 | 429.44 |  | <.001 |
| Gain |  |  |  |  |  |  |
|  | Intercept | 1 | 96 | 160445.32 |  | <.001 |
|  | Diagnostic Group | 3 | 96 | 0.635 | 0.174 | .594 |
|  | Regression Residual | 1 | 96 | 522.42 |  | <.001 |
| Gain variability | |  |  |  |  |  |
|  | Intercept | 1 | 96 | 8674.85 |  | <.001 |
|  | Diagnostic Group | 3 | 96 | 0.83 | 0.199 | .482 |
|  | Regression Residual | 1 | 96 | 1044.60 |  | <.001 |
| FEP |  |  |  |  |  |  |
|  | Intercept | 1 | 96 | 886510.67 |  | <.001 |
|  | Diagnostic Group | 3 | 96 | 0.99 | 0.217 | .398 |
|  | Regression Residual | 1 | 96 | 1111.93 |  | <.001 |
| FEP variability | |  |  |  |  |  |
|  | Intercept | 1 | 96 | 9390.42 |  | <.001 |
|  | Diagnostic Group | 3 | 96 | 0.94 | 0.211 | .427 |
|  | Regression Residual | 1 | 96 | 1663.60 |  | <.001 |

*Note.* Results for winsorised data presented*. df_Num_* = numerator degrees of freedom. *df_Den_* = denominator degrees of freedom. *F* = linear mixed effects model *F*-statistic. *d* = Cohen’s *d*. *p* = probability value for the F statistic -value. VGS = visually guided saccade. FEP = final eye position.

**Supplementary Material L**

**Kansas Cohort Confirmatory Dataset**

**Supplementary** **Table L1.** *Kansas Cohort: Sample characteristics for autism and Neurotypical groups.*

|  | Autism | NT | Whole sample |
| --- | --- | --- | --- |
|  | *n =* 29 | *n =* 41 | *N =* 70 |
| Child age (years) | 13.00 (2.33) | 13.00 (3.15) | 13.00 (2.82) |
| Child sex |  |  |  |
| Male | 22 | 23 | 45 |
| Female | 7 | 18 | 25 |
| FSIQ-4^a^ | 109.47 (9.89) | 114.41 (12.54) | 112.09 (11.47) |
| FSIQ-2^b^ | 109.89 (16.59) | 101.67 (20.23) | 107.83 (16.98) |
| ADOS-2^c^ | 7.68 (3.68) | NA | 7.68 (3.68) |
| SCQ^d^ | 18.12 (5.62) | 2.90 (2.91) | 11.36 (9.073) |

*Note.* Values = Mean (SD). ADHD = Attention Deficit/Hyperactivity Disorder. Autism = Autism Spectrum Disorder. Autism+ADHD = Co-Occurring Attention Deficit/Hyperactivity Disorder And Autism Spectrum Disorder. NT = Neurotypical. Child sex = frequency (percentage). FSIQ-4 = Full Scale Intelligence Quotient. ADOS-2 = Autism Diagnostic Observation Schedule – Second Edition Social Affect + Restrictive And Repetitive Behavior Total Score. SCQ = Social Communication Questionnaire Total Score. NA = Not Applicable as no data were collected for this group. ^a^*n* = 31. ^b^*n* = 12. ^c^*n* = 19. ^d^ *n* = 45.

## Supplementary Table L2. *Kansas Cohort: Means and Standard Deviations for the Oculomotor Function Outcomes Across Diagnostic Group and for Males and Females*

| *M (SD)* | Autism  *n =* 29 | Neurotypical  *n =* 41 |
| --- | --- | --- |
| **VGS Task^a^** |  |  |
| Relative time to peak velocity | 0.58 (0.23) | 0.57 (0.20) |
| Gain | 0.97 (0.14) | 0.94 (0.13) |
| Gain variability | 0.42 (0.12) | 0.35 (0.13) |
| FEP | 1.00 (0.08) | 0.99 (0.05) |
| FEP variability | 0.16 (0.08) | 0.11 (0.06) |
| First Saccade Latency^b^ | 448.45 (263.63) | 428 (249.37) |
| **Anti-saccade Task** |  |  |
| Number of directional errors | 27.31 (10.75) | 20.62 (9.78) |
| Number of anticipatory saccades | 4.34 (3.73) | 2.98 (3.25) |

*Note.* FEP = final eye position. ^a^*n* = 23 Autism, *n* = 36 Neurotypical participants completed the VGS Task. ^b^Reported for descriptive purposes only – not included in main analyses.

## Supplementary Table L3. *Kansas Cohort: Medication Use by Diagnostic Group*

| Medication | Autism | Neurotypical |
| --- | --- | --- |
| **CNS Stimulants** |  |  |
| Adderall | 0 (0.00%) | 0 (0.00%) |
| Concerta | 0 (0.00%) | 0 (0.00%) |
| Focalin | 0 (0.00%) | 0 (0.00%) |
| Metadate | 0 (0.00%) | 0 (0.00%) |
| Ritalin | 0 (0.00%) | 0 (0.00%) |
| Vyvanse | 1 (3.45%) | 0 (0.00%) |
| **Non-Stimulants** |  |  |
| Intuniv/Guanfacine | 3 (10.34%) | 0 (0.00%) |
| Strattera | 0 (0.00%) | 0 (0.00%) |
| Clonidine | 0 (0.00%) | 0 (0.00%) |
| **Atypical Antipsychotics** |  |  |
| Abilify | 0 (0.00%) | 0 (0.00%) |
| Risperdal | 1 (3.45%) | 0 (0.00%) |
| **Antidepressants** |  |  |
| Celexa | 0 (0.00%) | 0 (0.00%) |
| Cymbalta | 0 (0.00%) | 0 (0.00%) |
| Effexor | 0 (0.00%) | 0 (0.00%) |
| Lexapro | 1 (3.45%) | 0 (0.00%) |
| Mirtazapine | 0 (0.00%) | 0 (0.00%) |
| Prozac | 0 (0.00%) | 0 (0.00%) |
| Trazodone | 0 (0.00%) | 0 (0.00%) |
| Zoloft | 1 (3.45%) | 0 (0.00%) |
| **Anticonvulsants** |  |  |
| Depacon/Depakote/Depakene | 1 (3.45%) | 0 (0.00%) |
| **Hormones** |  |  |
| Melatonin | 5 (17.24%) | 0 (0.00%) |
| Norditropin | 1 (3.45%) | 0 (0.00%) |
| **Anticholinergic** |  |  |
| Oxybutinin | 0 (0.00%) | 0 (0.00%) |
| **Opiod** |  |  |
| Oxycodone | 1 (3.45%) | 0 (0.00%) |

*Note.* ADHD = attention deficit/hyperactivity disorder. Autism = autism spectrum disorder. Autism+ADHD = co-occurring attention deficit/hyperactivity disorder and autism spectrum disorder. CNS = Central Nervous System. Medication categories are not mutually exclusive, the same children may be counted for more than one. Percentages are column percentages.

## Supplementary Table L4. *Kansas Cohort: Linear Mixed Effects Model Fixed Effects Statistics for Diagnostic Group, and Regression Residual of Age and Sex*

| Oculomotor Measures | | *df_Num_* | *df_Den_* | *F* | *d* | *p* |
| --- | --- | --- | --- | --- | --- | --- |
| **VGS Task** | |  |  |  |  |  |
| Relative time to peak velocity | |  |  |  |  |  |
|  | Intercept | 1 | 55 | 5451.38 |  | <.001 |
|  | Diagnostic Group | 1 | 55 | 4.51 | 0.398 | .036 |
|  | Regression Residual | 1 | 55 | 682.48 |  | <.001 |
| Gain |  |  |  |  |  |  |
|  | Intercept | 1 | 55 | 20031.38 |  | <.001 |
|  | Diagnostic Group | 1 | 55 | 1.22 | 0.207 | .273 |
|  | Regression Residual | 1 | 55 | 364.18 |  | <.001 |
| Gain variability | |  |  |  |  |  |
|  | Intercept | 1 | 55 | 2677.891 |  | <.001 |
|  | Diagnostic Group | 1 | 55 | 2.69 | 0.307 | .107 |
|  | Regression Residual | 1 | 55 | 279.23 |  | <.001 |
| FEP |  |  |  |  |  |  |
|  | Intercept | 1 | 55 | 107120.81 |  | <.001 |
|  | Diagnostic Group | 1 | 55 | 0.19 | 0.082 | .669 |
|  | Regression Residual | 1 | 55 | 365.43 |  | <.001 |
| FEP variability | |  |  |  |  |  |
|  | Intercept | 1 | 55 | 259.20 |  | <.001 |
|  | Diagnostic Group | 1 | 55 | 5.25 | 0.429 | .026 |
|  | Regression Residual | 1 | 55 | 7.14 |  | <.001 |
| **Anti-saccade Task**  Number of directional errors | |  |  |  |  |  |
|  |  |  |  |  |  |  |
|  | Intercept | 1 | 55 | 307.29 |  | <.001 |
|  | **Diagnostic Group** | **1** | **55** | **5.81** | **0.452** | **.019** |
|  | Regression Residual | 1 | 55 | 1.75 |  | .192 |
| Number of anticipatory saccades | |  |  |  |  |  |
|  | Intercept | 1 | 67 | 716.64 |  | <.001 |
|  | Diagnostic Group | 1 | 67 | 0.17 | 0.077 | .680 |
|  | Regression Residual | 1 | 67 | 597.73 |  | <.001 |

*Note. df_Num_* = numerator degrees of freedom. *df_Den_* = denominator degrees of freedom. *F* = linear mixed effects model *F*-statistic. *p* = *p*-value. VGS = visually guided saccade. FEP = final eye position. **Bold** = statistically significant main effect. The Benjamini Hochberg corrected alpha value for Number of directional errors is *p* < .021; FEP variability is *p* < .023;  Relative time to peak velocity is *p* < .024.

## Supplementary Table L5. *Kansas Cohort: Linear Mixed Effects Model Diagnostic Group Pairwise Comparisons*

|  | Oculomotor Measures | | | Mean Diff. | Std. Error | | *df* | | | *d* | | *p* | | 95% CI | |  |
| --- | --- | --- | --- | --- | --- | --- | --- | --- | --- | --- | --- | --- | --- | --- | --- | --- |
|  |  |  |  |  |  |  |  |  |  |  | |  |  | Lower | Upper |  |
| Relative time to peak velocity | | | | | |  | |  |  | |  | |  | |  |  |
| NT | | Autism | .035 | | | 0.016 | | 55 | 0.602 | | 0.027 | | 0.004 | | 0.067 |  |
| Gain | | |  | | |  | |  |  | |  | |  | |  |  |
| NT | | Autism | 0.011 | | | 0.014 | | 55 | 0.217 | | 0.418 | | -0.016 | | 0.038 |  |
| Gain Variability | |  |  | | |  | |  |  | |  | |  | |  |  |
| NT | | Autism | 0.015 | | | 0.015 | | 55 | 0.270 | | 0.315 | | -0.015 | | 0.046 |  |
| FEP | |  |  | | |  | |  |  | |  | |  | |  |  |
| NT | | Autism | 0.003 | | | 0.006 | | 55 | 0.125 | | 0.669 | | -0.010 | | 0.015 |  |
| FEP Variability | |  |  | | |  | |  |  | |  | |  | |  |  |
| NT | | Autism | -.038 | | | 0.017 | | 55 | -0.624 | | 0.026 | | -0.072 | | -0.005 |  |
| Number of Directional Errors | | |  | | |  | |  |  | |  | |  | |  |  |
| **NT** | | **Autism** | **-6.638** | | | **2.755** | | **55** | **-0.624** | | **0.019** | | **-12.159** | | **-1.117** |  |
| Number of Anticipatory Saccades | | |  | | |  | |  |  | |  | |  | |  |  |
| NT | | Autism | -0.203 | | | 0.290 | | 67 | -0.171 | | 0.487 | | -0.781 | | 0.375 |  |

*Note.* Mean diff = mean difference. Std. error = standard error. df = degrees of freedom. *d* = Cohen’s *d*. *p* = *p*-value. 95% CI = 95% confidence interval. Lower = lower bound. Upper = upper bound. NT = Neurotypical. Autism = autism spectrum disorder. FEP = final eye position. ^a^Log_10_ transformation. **Bold** = statistically significant after Bonferroni correction.
